# *Akkermansia muciniphila* exacerbates food allergy in fibre-deprived mice

**DOI:** 10.1038/s41564-023-01464-1

**Published:** 2023-09-11

**Authors:** Amy Parrish, Marie Boudaud, Erica T. Grant, Stéphanie Willieme, Mareike Neumann, Mathis Wolter, Sophie Z. Craig, Alessandro De Sciscio, Antonio Cosma, Oliver Hunewald, Markus Ollert, Mahesh S. Desai

**Affiliations:** 1 Department of Infection and Immunity, Luxembourg Institute of Health, Esch-sur-Alzette, Luxembourg; 2 Faculty of Science, Technology and Medicine, University of Luxembourg, Esch-sur-Alzette, Luxembourg; 3 National Cytometry Platform, Luxembourg Institute of Health, Esch-sur-Alzette, Luxembourg; 4 Odense Research Center for Anaphylaxis, Department of Dermatology and Allergy Center, Odense University Hospital, University of Southern Denmark, Odense, Denmark

**Keywords:** Microbiome, Allergy

## Abstract

Alterations in the gut microbiome, including diet-driven changes, are linked to the rising prevalence of food allergy. However, little is known about how specific gut bacteria trigger the breakdown of oral tolerance. Here we show that depriving specific-pathogen-free mice of dietary fibre leads to a gut microbiota signature with increases in the mucin-degrading bacterium *Akkermansia muciniphila*. This signature is associated with intestinal barrier dysfunction, increased expression of type 1 and 2 cytokines and IgE-coated commensals in the colon, which result in an exacerbated allergic reaction to food allergens, ovalbumin and peanut. To demonstrate the causal role of *A. muciniphila*, we employed a tractable synthetic human gut microbiota in gnotobiotic mice. The presence of *A. muciniphila* within the microbiota, combined with fibre deprivation, resulted in stronger anti-commensal IgE coating and innate type-2 immune responses, which worsened symptoms of food allergy. Our study provides important insights into how gut microbes can regulate immune pathways of food allergy in a diet-dependent manner.

## Abstract (web summary)

The gut commensal *Akkermansia muciniphila* regulates food allergy symptoms in a diet-dependent manner in mice.

## Main

As the Western world continues to see rising rates of food allergy^1,2^ that cannot be explained solely by genetics^3,4^, identifying mechanisms of sensitization that are driven by environmental factors has become increasingly important. The gut microbiota has been shown to play an important role in food allergy by inducing protection against sensitization^5–7^. This is evident from studies in germ-free (GF) mice that develop severe anaphylaxis, which can be dampened with the colonization of single microbes or complex communities of bacteria^7^. Moreover, mouse studies utilizing high-fat^8^ or low-fibre^9^ diets have shown that diet-mediated changes in the gut microbiota are associated with food allergy sensitization. Nevertheless, little is known about the causal links between food allergy sensitization and specific members of the microbiome, and how certain microbes regulate allergic responses.

Sensitization in atopic diseases begins at barrier sites and, in the context of food allergy, a functional epithelial barrier has been shown to be crucial in preventing sensitization and promoting oral tolerance^3^. An important part of the intestinal mucosal barrier is the mucus layer, which serves as a first line of defence in the gut to prevent microbial invasion but allow nutrient absorption^10,11^. Although gut exposure to allergens is understood to induce immunotolerance in a healthy gut, tolerance breakdown may occur when the mucus layer is genetically ablated, as shown in *Muc2*^*−/−*^ mice that lack the gene for *O*-glycosylated mucin-2 (MUC2), the main constituent of the mucus layer^12^. Induction of tolerance in the gut through regulatory T cells is mediated by the sensing of MUC2 by local dendritic cells (DC), highlighting the importance of the mucus barrier in food allergy^12,13^.

Diet is an important environmental factor that affects the mucus barrier through the microbiota because: (1) dietary fibre deprivation increases deterioration of the colonic mucus layer by mucolytic gut bacteria^14,15^, and (2) Western-style diet increases the permeability of the colonic mucus layer^16^. Fibre-deprived microbiota-mediated mucus barrier dysfunction is reflected in enhanced susceptibility to infection by *Citrobacter rodentium*, a pathogen that must cross the mucus layer to reach the gut epithelium and initiate mucosal infection^14,17^. Nevertheless, little is known about how diet-mediated barrier dysfunction together with increased activities of specific mucin-degrading microbes might impact oral tolerance.

In this Article, we hypothesized that dietary fibre deprivation, which can drive a mixed type 1 and 2 immune landscape together with an increase in mucin-degrading gut microbes, predisposes the host to enhanced allergen sensitization. Using two experimental allergens, ovalbumin and peanut, we establish a causal link between *Akkermansia muciniphila* and allergic immune responses by promoting type-2 immunity.

## Results

### Fibre deprivation leads to microbiota-mediated colonic barrier dysfunction

**Fig. 1:**
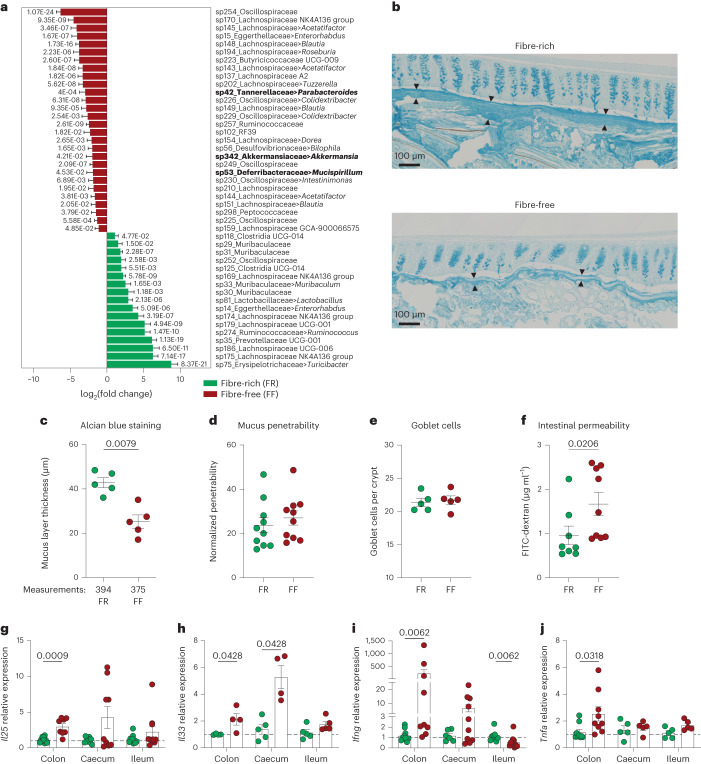
Fibre deprivation leads to microbiota-mediated mucosal barrier breakdown. Mice were fed an FR (green dots) or an FF (red dots) diet for 40 days. **a**, Differential abundance analysis of taxa in the faeces of FR-fed mice (green bar) versus FF-fed mice (red bar) (*n* = 10 mice per group, 5 mice per cage, Wald test, *P* values adjusted using the Benjamini–Hochberg method, features with less than 1 count on average across all samples were excluded). Data are presented as mean ± s.e.m. Taxa in bold depict known/potential mucin-degrading bacteria; for *Akkermansia*, ~1.78-fold increase in FF- compared with FR-fed mice (change from average relative abundance of 1.7% to 5.7%). **b**, Representative images of Alcian blue-stained longitudinal colonic sections of mice fed an FR or FF diet for 40 days. Black arrowheads indicate the edges of the mucus layer (*n* = 5 mice per group) **c**, Mucus layer thickness measured on colonic section stained with Alcian blue. Each dot is the average of several measurements from one animal (*n* = 5 mice per group, two-tailed Mann–Whitney test). The average number of measurements per mouse is indicated on the *X* axis. **d**, Colonic mucus layer penetrability to 1-μm-sized beads. Each dot is an average of 4–7 measurements from one animal (*n* = 10 mice per group, two independent experiments). **e**, Goblet cell counts per crypt. Each dot is the average of multiple measurements from one animal (*n* = 5 mice per group). **f**, Intestinal permeability to FITC-dextran (*n* = 8–9 mice per group, two independent experiments, two-tailed Mann–Whitney test). **g**–**j**, Relative transcript levels of *Il25* (**g**), *Il33* (**h**), *Ifng* (**i**) and *Tnfa* (**j**) mRNA in the colon, caecum and ileum. Expression levels were normalized to the FR group, independently for each tissue (*n* = 5–10 mice per group, two independent experiments, multiple Mann–Whitney, *P* values adjusted using the Benjamini–Hochberg method). Each dot represents one mouse. All dot plots are represented with mean ± s.e.m.

We fed specific-pathogen-free (SPF) BALB/c mice for 40 days on a fibre-rich (FR) (standard laboratory chow) or a fibre-free (FF) diet. In line with our previously published results in gnotobiotic mice with a characterized 14-member synthetic human gut microbiota (14SM)^14^, the microbiota of FF-fed mice exhibited increased relative abundances of bacteria belonging to the known mucin-specialist genus *Akkermansia* (Fig. 1a). In addition, other putative mucin-degrading species (Fig. 1a, shown in bold), such as *Mucispirillum*^18^ and *Parabacteroides*^19^, were also increased in the FF-fed mice.

**Extended Data Fig. 1:**
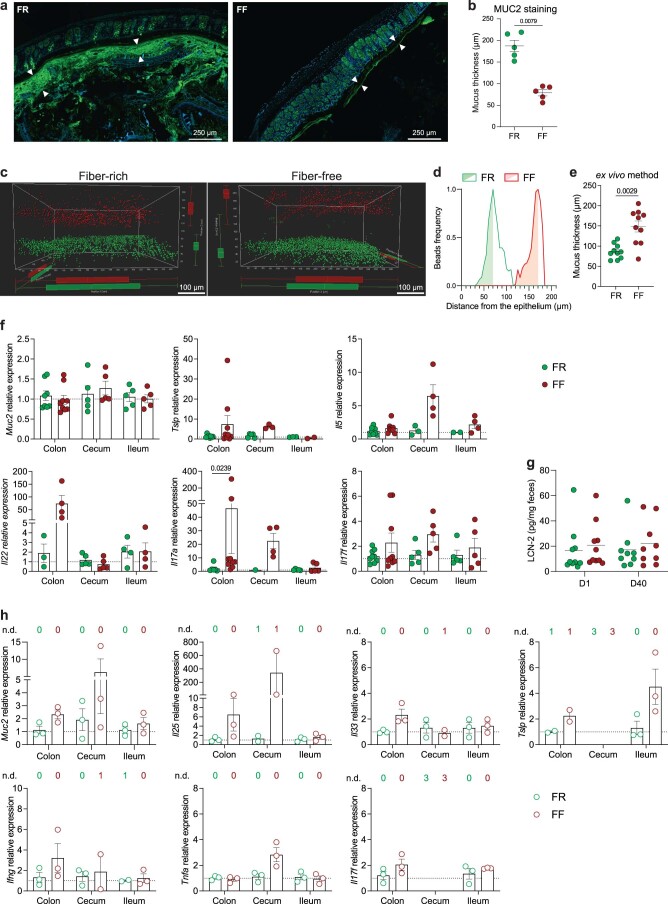
**a**, Representative immunofluorescence images of distal colon sections stained with α-Muc2 (green) and DAPI (blue). White arrow heads indicate the edges of the mucus layer. **b**, Mucus thickness measured on colonic section stained with α-Muc2. Each dot is the average of several measurements from one animal (*n* = 5 mice per group, two-tailed Mann-Whitney test). **c**, Representative images of distal colonic tissues showing 1-μm-sized beads (red dots) laying on top of the epithelium (green dots) using an *ex situ ex vivo* method. The space between the beads and the epithelium exemplify the unpenetrable mucus layer. **d**, Analysis of the images presented in b showing the frequency of beads as a function of the distance of the epithelium for the FR-fed (green line) and the FF-fed (red line) sample. The penetration of the beads into the mucus is represented and quantified as the area under the curve to the peak (colored area under the curve). **e**, Colonic mucus thickness measured *ex vivo* as the distance of 1-μm-sized beads peak from the epithelium (as shown in **d**). Each dot is the average of 4–7 measurements from one animal (*n* = 10 mice per group, two-tailed Mann-Whitney test). **f**, Relative transcript levels of *Muc2, Tslp, Il5, Il22, Il17a and Il17f* in the colon, cecum, and ileum of SPF mice fed a FR (green) or a FF (red) diet. Expression levels were normalized to the FR group, independently for each tissue (*n* = 5–10 mice per group, two independent experiments, multiple Mann–Whitney test, *P* values adjusted using the Benjamini-Hochberg method). **g**, Fecal lipocalin-2 (LCN-2) measured at the beginning (D1) and at the end (D40) of the 40-day feeding period (*n* = 9–10). **h**, Relative transcript levels of selected cytokine mRNA in the colon, cecum and ileum of germfree mice. Transcript levels were normalized to the FR group, independently for each tissue. Numbers above dot plots represent the number of samples in which transcripts were not detected (n.d.) (*n* = 3 mice per group). All dot plots are represented with mean +/− SEM.

In line with previous findings^14,15,17^, the FF-fed mice exhibited approximately 2-fold reduction in colonic mucus thickness (Fig. 1b,c and Extended Data Fig. 1a,b). Using an ex vivo ex situ method to assess the penetrability of the mucus on dissected colonic tissues^20^, we found that the mucus displayed similar normalized penetrability to bacteria-sized fluorescent beads between the two diets (Fig. 1d and Extended Data Fig. 1c,d). Intriguingly, with this method, the overall mucus thickness, determined as the distance of the beads from the epithelium was significantly higher in the FF-fed mice, suggesting changes in mucus architecture under fibre deprivation (Extended Data Fig. 1c–e). Fibre deprivation did not affect the number of goblet cells per crypt (Fig. 1e), suggesting that mucus production is not affected by diet. However, despite a similar penetrability of the colonic mucus to bacteria-sized beads (Fig. 1d), the overall intestinal permeability to FITC-dextran was increased in FF-fed mice (Fig. 1f), suggesting a higher permeability of the overall gut mucosal barrier to luminal antigens during fibre deprivation.

The relative transcript levels of the epithelial-derived cytokines^21^, IL-25, IL-33 and TSLP, were increased in the colon and/or in the caecum, but not in the ileum, in FF-fed mice (Fig. 1g,h and Extended Data Fig. 1f). Consistently, the *Il5* transcript tended to be increased in FF caecal tissues (Extended Data Fig. 1f). In addition, FF-fed mice displayed increased levels of type 1 and 17 cytokine transcripts, *Ifng*, *Tnfa*, *Il22* and *Il17a* in the colon (Fig. 1i,j and Extended Data Fig. 1f). Although the higher transriptional levels of inflammatory cytokines were not reflected in the faecal level of the neutrophilic inflammatory marker lipocalin (LCN-2) (Extended Data Fig. 1g), these data reveal an inflammatory response reflective of a gut barrier impairment that is specific to the lower intestinal tract (caecum and colon) under fibre deprivation. Intriguingly, tissues from fibre-deprived GF mice seemed to have slightly higher levels of *Il25* and *Il33* in the colon compared with FR tissues, while *Tslp* expression was barely detectable and, *Ifng* and *Tnfa* remained at low levels compared with those from SPF FF-fed mice (Extended Data Fig. 1h), suggesting that FF diet alone in the absence of microbiota contributes to some of the immune changes.

### Colonic barrier dysfunction is associated with local type-2 inflammation

**Fig. 2:**
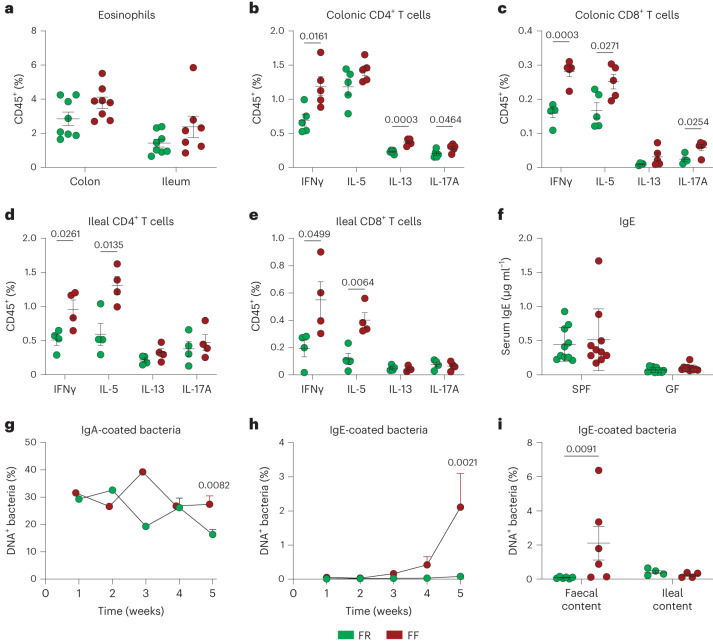
Fibre deprivation induces type-2 immune changes in the intestinal mucosa. Mice were fed an FR (green dots) or an FF (red dots) diet for 40 d. **a**, Eosinophil cell frequencies among CD45^+^ single cells in the colonic and ileal lamina propria (*n* = 7–8 mice per group, two independent experiments, two-way ANOVA, organ effect *P* = 0.0018, diet effect *P* = 0.0281). **b**–**e**, Frequencies of cytokine-expressing colonic CD4^+^ (**b**) and CD8^+^ (**c**) T cells and ileal CD4^+^ (**d**) and CD8^+^ (**e**) T cells among CD45^+^ cells (*n* = 4–5 mice per group, multiple unpaired *t*-test). **f**, Serum titres of IgE (*n* = 9–10 mice per group, two independent experiments, two-way ANOVA). **g**,**h**, Frequencies of faecal IgA-coated (**g**) and IgE-coated (**h**) bacteria over 5 weeks of feeding on FR or FF diet (*n* = 1–5 mice per group, two-way ANOVA, *P* values adjusted using the Benjamini–Hochberg method). **i**, Frequencies of faecal and ileal IgE-coated bacteria after 40 d of feeding (*n* = 4–6 per group, multiple Mann–Whitney test, *P* values adjusted using the Benjamini–Hochberg method). All dot plots are represented with mean ± s.e.m.

**Extended Data Fig. 2:**
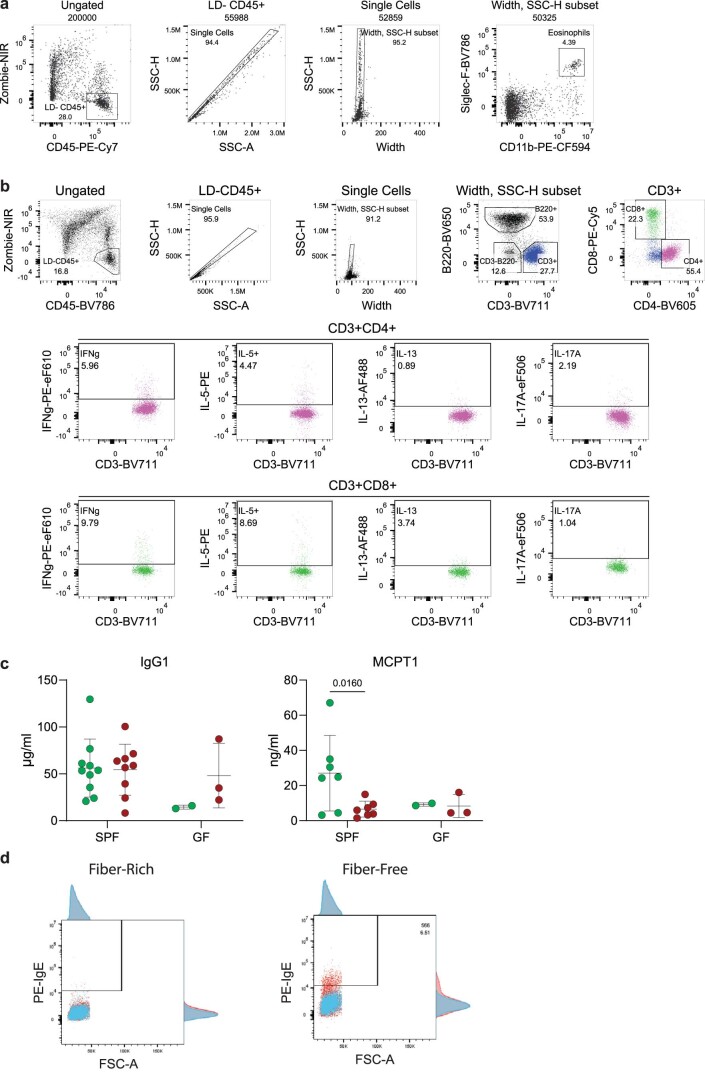
**a**, Gating strategy for eosinophils, defined as Siglec-F^+^CD11b^+^ singlets. **b**, Gating strategy for cytokine-expressing CD4^+^ and CD8^+^ T lymphocytes. **c**, Serum titers of IgG1 and mouse mucosal mast cell protease 1 (MCPT1) (*n* = 2–10, two-way ANOVA, *P* values adjusted using the Benjamini-Hochberg method). **d**, Representative plots for IgE-coated bacteria in fiber-rich and fiber-free fed mice. Each sample was stained with an α-IgE antibody (red) or an isotype control (blue).

Using mass cytometry by time-of-flight (CyTOF)-based broad immunophenotyping, our recently prepublished data showed that FF-fed mice exhibit a biased immune landscape in the colonic lamina propria (cLP), with an increase in type-2 inflammatory cells such as ILC2, mast cells and activated Th2 cells^22^. Targeted fluorescence-activated cell sorting (FACS) analysis revealed a higher frequency of eosinophils in the colon and ileum of FF-fed mice (Fig. 2a, two-way analysis of variance (ANOVA), diet effect *P* = 0.0281; and Extended Data Fig. 2a), along with a higher frequency of IL-5-producing CD4^+^ and/or CD8^+^ T cells in the colon and ileum (Fig. 2b–e and Extended Data Fig. 2b). Consistent with an overall increase in activated CD69^+^ T cells^22^, FF-fed mice had also more IFNγ-, IL-13- and IL-17-producing T cells in the colon (Fig. 2b,c), while in the ileum, only IFNγ-producing T cells were enriched in addition to IL-5-producing cells (Fig. 2d,e). The titres of serum IgE and IgG1 were not different between the two dietary groups, although they were higher in SPF than in GF mice (Fig. 2f and Extended Data Fig. 2c). However, the titres of mast cell protease 1 (MCPT1) were reduced in the FF-fed SPF mice compared with FR-fed SPF mice, but not in the GF control groups (Extended Data Fig. 2c). On the basis of the serum data, these results show that FF-fed mice develop a type-2 immune profile that is restricted to the gut.

To determine whether the increased type-2 inflammation within the colon could be reflected in the anti-commensal antibody response, we analysed the IgA- and IgE coating of faecal bacteria over the 40-day feeding period. As expected, bacteria were mainly coated with IgA, with a detection of ~30% IgA-coated bacteria at the beginning of the timecourse and remaining relatively stable between the two diets over time (Fig. 2g). In contrast, IgE-coated bacteria were almost undetectable at the beginning of the experiment and increased over time in FF-fed mice only, reaching up to 2% of faecal bacteria at week 5 (Fig. 2h and Extended Data Fig. 2d). In contrast, ileal bacteria remained minimally coated with IgE in both groups (Fig. 2i). In line with the barrier breakdown profiles (Fig. 1), these results: (1) support a colon-specific, anti-commensal IgE response driven by FF diet; and (2) point to a possible mechanism of allergic sensitization through a more permeable intestinal barrier and a type-2 skewed immune landscape.

### Diet-driven barrier dysfunction is reflected in exacerbated food allergic responses

**Fig. 3:**
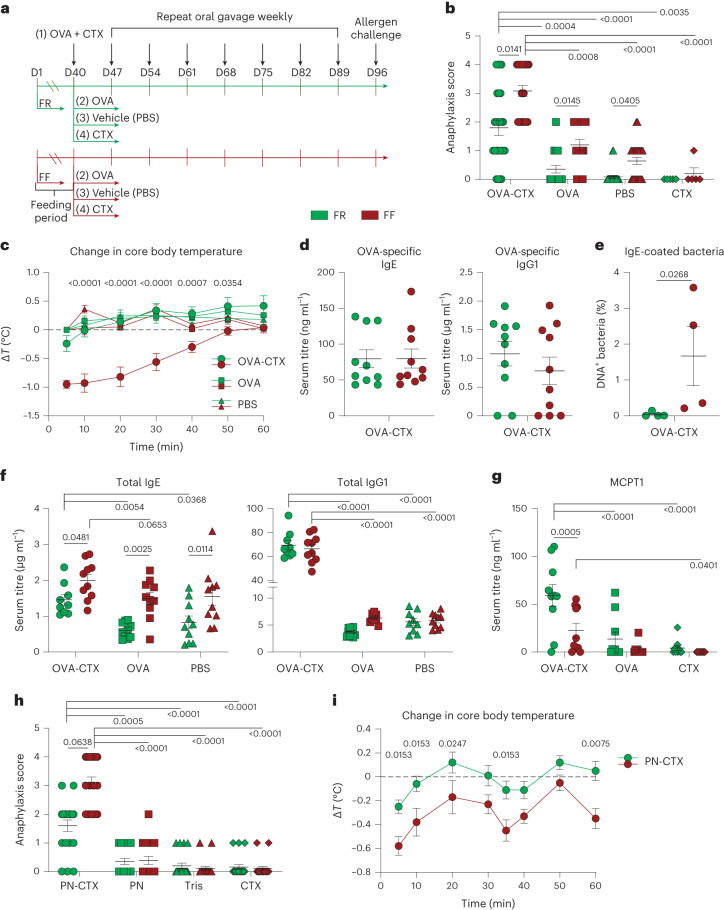
Fibre deprivation worsens food allergic responses. **a**, Schematic timeline of OVA sensitization with cholera toxin (CTX) as an adjuvant and control groups. **b**,**c**, Blinded symptom scores (**b**) and core body temperature (**c**) acquired at OVA challenge (*n* = 20 mice per group, two independent experiments, Kruskal–Wallis test (**b**) or two-way ANOVA (**c**), *P* values adjusted using the Benjamini–Hochberg method, between FR and FF among OVA-CTX mice). **d**, Serum titres of OVA-specific IgE and IgG1 (*n* = 10 mice per group, two-tailed unpaired *t*-tests). Note that the titres of both OVA-specific IgE and IgG1 for all other groups were below the limit of detection. **e**, Frequencies of IgE-coated bacteria in the colonic content of mice at the end of the experiment (*n* = 4 mice per group, two-sided Mann–Whitney test). **f**,**g**, Serum titres of total IgE and IgG1 (**f**) and mouse mast cell protease 1 (**g**, MCPT1) (*n* = 10 mice per group, two independent experiments, two-way ANOVA, *P* values adjusted using the Benjamini–Hochberg method). **h**,**i**, Blinded symptom scores (**h**) and core body temperature (**i**) acquired at peanut (PN) challenge (*n* = 20 mice per group, two independent experiments, Kruskal–Wallis test (**h**) or two-way ANOVA (**i**), *P* values adjusted using the Benjamini–Hochberg method, between FR and FF among PN-CTX mice). All dot plots are represented with mean ± s.e.m.

**Extended Data Fig. 3:**
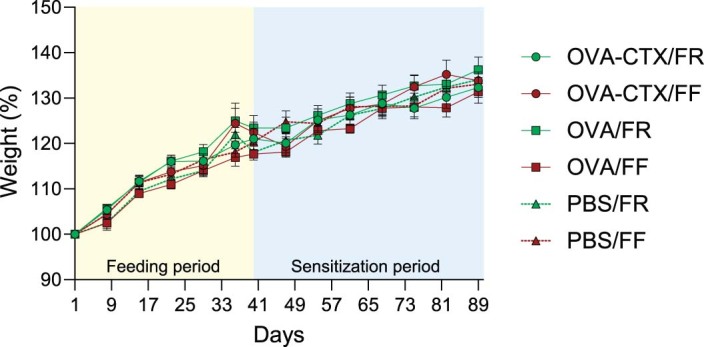
Weight curves of the mice over the 40-day feeding period and the sensitization period to OVA-CTX (n = 5–10, mixed-effects model with matched values per mice, time effect ***P*** < 0.0001).

We further hypothesized that the FF-fed mice may be rendered more susceptible to food allergen sensitization via the oral route. To test this hypothesis, mice were sensitized with ovalbumin (OVA) via the gastro-intestinal route by gavaging the allergen along with cholera toxin (CTX) as an adjuvant to promote the breakdown of oral tolerance and induce allergic reactions with moderate severity as previously decribed (Fig. 3a)^23^. The FF diet or sensitization did not affect the weight gain of mice over time (Extended Data Fig. 3). Upon allergen challenge, FF-fed sensitized (OVA-CTX) mice exhibited more severe anaphylactic symptom scores (Fig. 3b) and a greater drop in core body temperature (Fig. 3c) compared with FR-fed sensitized (OVA-CTX) mice. Interestingly, FF-fed mice that were exposed solely to the allergen without adjuvant (OVA) or with PBS only also exhibited slightly increased symptom scores compared with their FR-fed controls, suggesting an effect of fibre deprivation on mild allergen-independent symptoms (Fig. 3b). Nevertheless, OVA-specific IgE and IgG1 were detectable only in the serum of OVA-CTX mice, confirming the model of adjuvant-mediated breakdown of oral tolerance^23^ (Fig. 3d). Fibre deprivation did not affect the level of sensitization as defined by the serum titres of OVA-specific IgE and IgG1 (Fig. 3d). Sensitization did not increase the overall frequency of IgE-coated bacteria (Fig. 3e) compared to FF-fed unsensitized mice (Fig. 2i), signifying that the increased IgE coating occurs independently of allergen sensitization. In addition, our data show an increase in total IgE and IgG1 in OVA-CTX-sensitized mice compared with unsensitized mice, and higher total IgE in FF-fed mice compared with FR-fed mice regardless of treatment (Fig. 3f). Of note, while the total IgE titres did not differ between FR- and FF-fed mice after 40 d of feeding (Fig. 2f), unsensitized mice treated with PBS showed higher titres when fed FF diet compared with FR diet (Fig. 3f), suggesting that a feeding period longer than 40 d may result in an atopic phenotype. Finally, although serum MCPT1 titres were increased in OVA-CTX sensitized mice compared with OVA- or CTX-treated mice, they remained lower in FF-fed sensitized mice compared with FR-fed sensitized mice, suggesting that cellular pathways alternative to mast cells are involved in worsening food allergy caused by fibre deprivation (Fig. 3g).

**Fig. 4:**
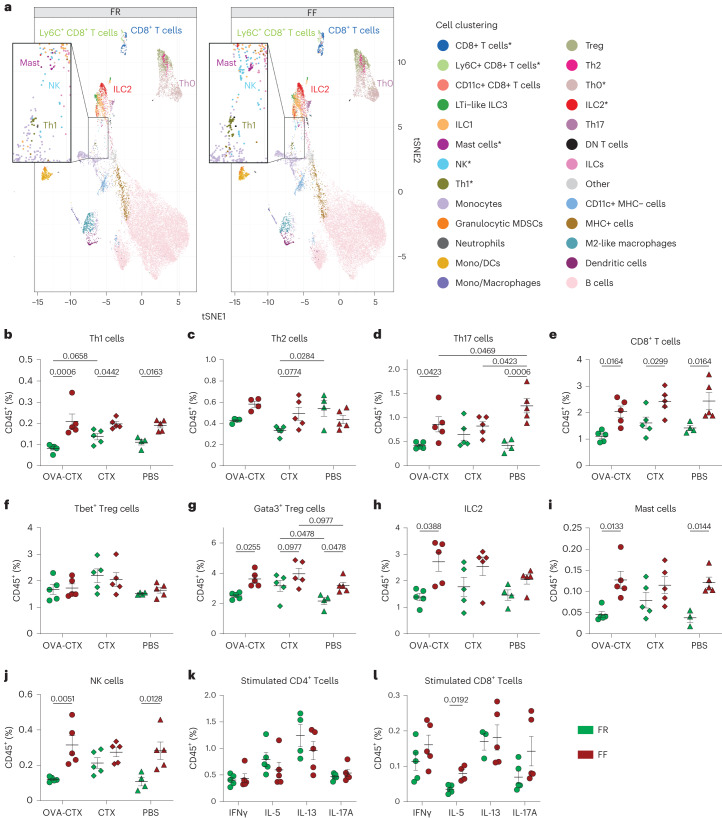
Fibre deprivation increases colonic inflammation induced by food allergen sensitization. **a**, UMAP of the main populations identified using FlowSOM among CD45^+^ cells from the colonic lamina propria of FR- and FF-fed OVA-CTX-sensitized SPF mice 24 h after challenge (*n* = 5 per group, multiple Mann–Whitney test, *P < 0.05). **b**–**j**, Frequencies of indicated cell populations among CD45^+^ cells from the colonic lamina propria of FR-fed (green) and FF-fed (red) mice (*n* = 5 mice per group, two-way ANOVA, unadjusted *P* values). **k**,**l**, Frequencies of cytokine-expressing CD4^+^ (**k**) and CD8^+^ (**l**) T cells among colonic CD45^+^ cells (*n* = 4–5 mice per group, multiple unpaired *t*-test, *P* values adjusted using the Benjamini–Hochberg method). All dot plots are represented with mean ± s.e.m.

**Extended Data Fig. 4:**
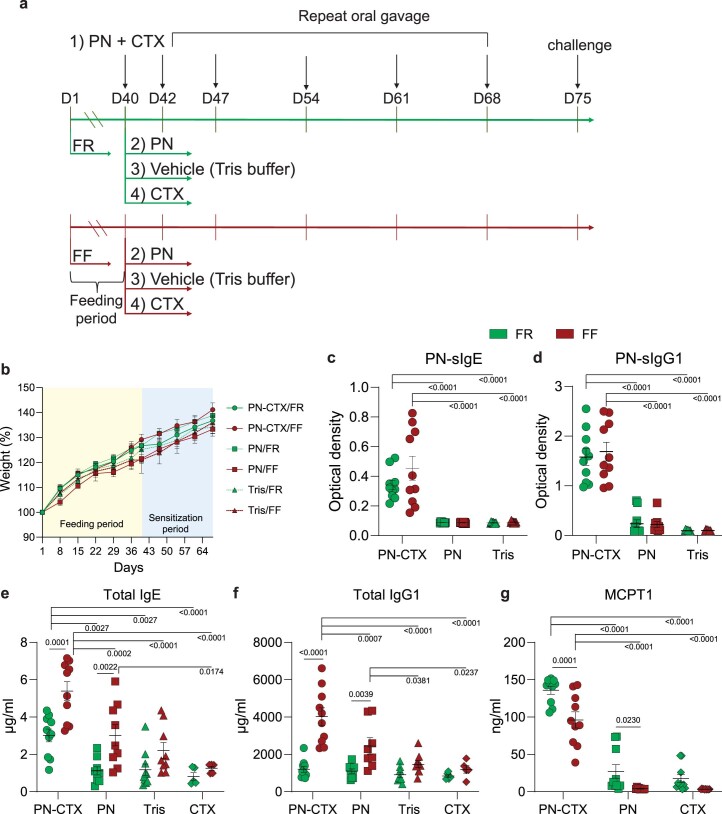
**a**, Schematic timeline for peanut protein (PN) sensitization with cholera toxin (CTX) as an adjuvant and control groups. Samples were collected 3 h after the challenge. **b**, Weight curves of the mice over the 40-day feeding period and the sensitization period to PN-CTX (n = 10, two independent experiments, mixed-effects model with matched values per mice, time effect *P* < 0.0001). **c–g**, Serum titers of PN-specific IgE (**c**, *n* = 9–10), PN-specific IgG1 (**d**, *n* = 9–10), total IgE (**e**, *n* = 5–10), total IgG1 (**f**, *n* = 5–10), and mouse mast cell protease 1 (**g**, MCPT1, *n* = 10). Two-way ANOVA, *P* values adjusted using the Benjamini-Hochberg method. Fiber-rich (FR, green), fiber-free (FF, red). All dot plots are represented with mean +/− SEM.

**Extended Data Fig. 5:**
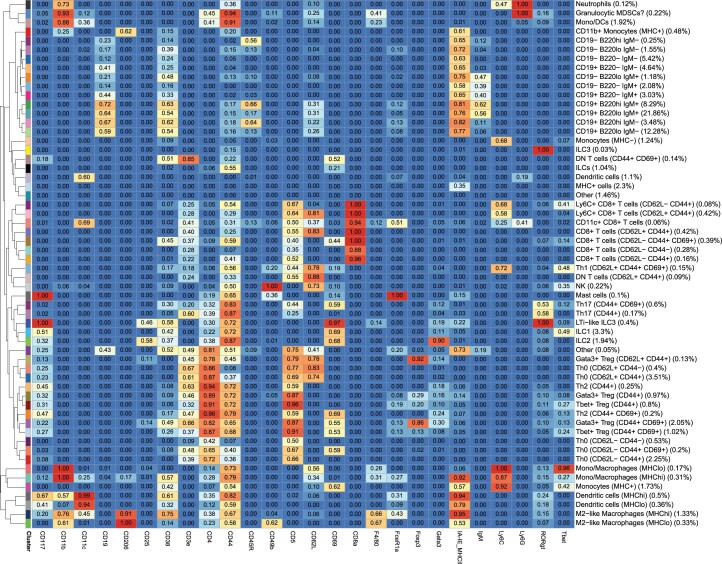
Heatmap of the 58 annotated clusters identified by FlowSOM from CyTOF analysis of colonic lamina propria cells of SPF mice 24 h after challenge. Numbers in the heatmap indicate the relative expression of each marker among cell populations. Percentages shown in parantheses represent frequencies of populations among CD45^+^ cells.

We next employed an additional allergen, peanut (PN) (Extended Data Fig. 4a). Neither the diet nor the sensitization affected the weight gain of the mice in the course of the experiment timeline (Extended Data Fig. 4b). FF-fed PN-sensitized (PN-CTX) mice showed more severe symptom scores and temperature drops compared with FR-fed sensitized (PN-CTX) mice (Fig. 3h,i). Although PN-specific IgE and IgG1 were similar between the two diet groups (Extended Data Fig. 4c,d), fibre deprivation increased total IgE and IgG1 levels induced by both PN-CTX or PN sensitization (Extended Data Fig. 4e,f). In addition, among PN-sensitized mice, FF-fed mice had decreased serum MCPT1 compared with FR-fed mice (Extended Data Fig. 4g), which was similar to the findings from OVA sensitization. As the results between the two food allergens (OVA and PN) were largely consistent, we elected to focus our attention on OVA sensitization for further immune characterizations using CyTOF (Fig. 4a and Extended Data Fig. 5).

Although the majority of immune cells in the cLP were B cells and regulatory T (Treg) cells (~60% and 5% of CD45^+^ cells, respectively), dietary fibre deprivation mostly affected the proportion of less frequent but inflammatory cells (Fig. 4a). Among OVA-CTX-sensitized mice, the FF diet increased the frequencies of type-2 cells (Th2, ILC2 and mast cells), as well as type-1 (Th1, NK, ILC1 and CD8+ T cells) and type-3 cells (Th17, ILC3) (Fig. 4b–j). Although the sensitization tended to decrease the frequencies of Th1 and Th17 cells in FR-fed and FF-fed mice, respectively, the effect of fibre deprivation was also seen in PBS control mice, suggesting a sensitization-independent effect of the diet on the immune landscape (Fig. 4b–j). In addition to inflammatory cells, Treg cells, particularly Gata3^+^ but not Tbet^+^, were more present in the cLP of FF-fed mice (Fig. 4f,g). Although typically described as type-1 inflammatory cells, CD8^+^ T cells have recently been highlighted for their role in food allergy^24^. Supporting a role in the fibre deprivation-increased sensitization shown here, IL-5-producing CD8^+^ T cells, but not CD4^+^ T cells, were enriched in the cLP of FF-fed OVA-CTX-sensitized mice (Fig. 4k,l).

### Allergic sensitization shifts the microbiome in a diet-dependent manner

**Fig. 5:**
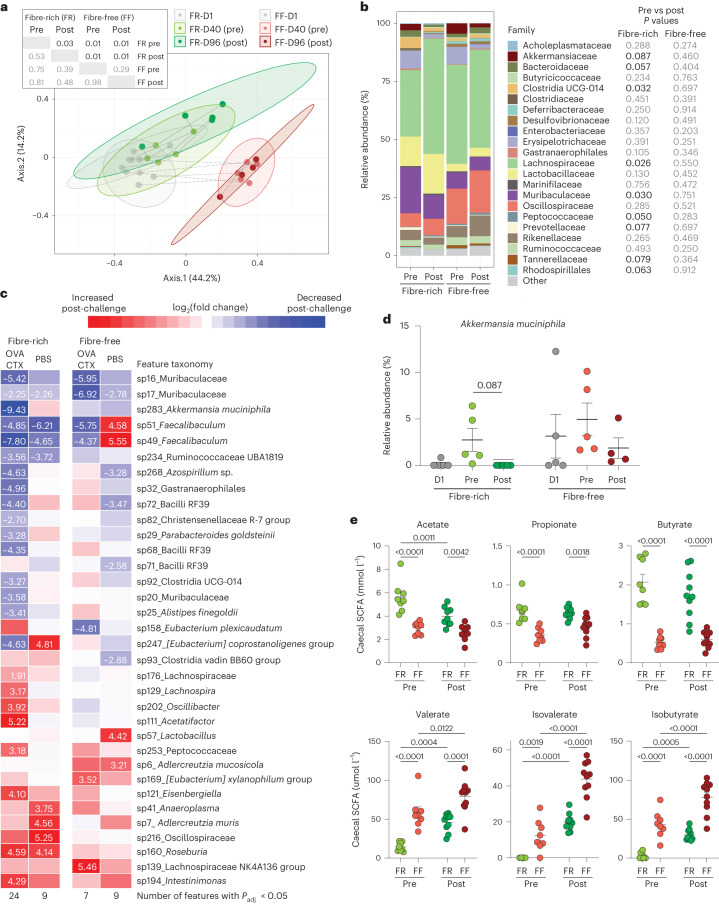
Food allergen sensitization affects the gut microbiota composition in a diet-dependent manner. **a**, PCoA plot of microbiome profiles using Bray–Curtis dissimilarity index for FR- or FF-fed mice at the beginning of the feeding period (D1), before sensitization (pre, D40) and after sensitization (post, D96). Inset table: *P* values from testing for heterogeneity of dispersion (left of diagonal) and distance between group centroids (right of diagonal). Data for D1 and D40 are shared with a previous study^22^. **b**, Family-level barplots of relative abundance pooled by diet group and time; *P* < 0.1 indicated with black text colour (*n* = 4–5, multiple paired *t*-tests, all comparisons non-significant after adjustment for multiple comparisons using the Benjamini–Hochberg method). **c**, Heat map of log_2_(fold change) of taxonomic features post-challenge (relative to pre-challenge) among FR- or FF-fed mice that were OVA-sensitized or received PBS as a control. Only those taxa with *P*_adj_ < 0.05 for at least one group are shown. At the bottom of the heat map, the number of features significantly affected by the challenge are listed for each group. **d**, Relative abundance of *A. muciniphila* from the first day of the experiment (D1), from before sensitization (pre) and after sensitization (post) in FR- and FF-fed mice (*n* = 4–5, two-way ANOVA, individual *P* < 0.05, non-significant after *P* values were adjusted using the Benjamini–Hochberg method). **e**, Concentrations of short- and branched-chain fatty acids in the caecal contents of mice before sensitization (pre) and after sensitization (post) (*n* = 8–10, two-way ANOVA, *P* values adjusted using the Benjamini–Hochberg method). All dot plots are represented with mean ± s.e.m.

A microbiome signature has been previously linked with paediatric food allergy^25^, characterized by increased abundance of the mucin degrader *Ruminococcus gnavus*^26^ and decreased abundance of several fibre-degrading and probiotic (that is, *Bifidobacterium longum*) species compared with healthy controls^25^. Nevertheless, it remains unclear whether these microbial changes occur before or as a consequence of allergic sensitization. Faecal microbiome profiles, as summarized by the Bray–Curtis dissimilarity index, were significantly shifted in FR-fed mice upon challenge, but not in FF-fed mice (Fig. 5a). Of note, the microbiome of mice differed mostly depending on the diet (Fig. 5a), suggesting that fibre deprivation itself already creates a stronger selective pressure on the microbiome composition than the sensitization process. Among FR-fed mice, barplots of the relative abundances at the family level revealed increases in the Lachnospiraceae and Peptococcaceae families, and decreases in the families Akkermansiaceae, Bacteroidaceae, Clostridia UCG-014, Muribaculaceae, Prevotellaceae, Tannerallaceae and the order Rhodospiralles due to allergic sensitization (Fig. 5b,c).

Differential analysis confirmed significant shifts for 24 features in FR-fed sensitized mice and 7 features in FF-fed sensitized mice (Fig. 5c). Of these, 3 features were significantly changed due to sensitization in FF-fed mice, but not in FR-fed mice, these features belonging either to the genus *Eubacterium* (sp158 and sp169) or to the family Lachnospiraceae (sp139) (Fig. 5c). In contrast, among the features that changed only in FR-fed mice following sensitization, *A. muciniphila* showed the largest shift (Fig. 5c). *A. muciniphila* abundance increased after the 40-day feeding period (pre) and decreased after sensitization (post) in both FR- and FF-fed mice (Fig. 5c). However, while this mucin-degrading bacterium was not detected after sensitization in FR-fed mice, the shift was not as evident in FF-fed mice, probably owing to the mucin-foraging niche created and supported by fibre deprivation (Fig. 5c,d). Consistently, the shift in microbial metabolism under fibre deprivation could be observed similarly before and after sensitization through reduced caecal concentrations of short-chain fatty acids (SCFAs) butyrate, propionate and acetate, and increased concentrations of valerate and protein-derived branched-chain fatty acids (BCFAs) isovalerate and isobutyrate (Fig. 5e). In addition, while BCFA titres increased over the sensitization period in both FR- and FF-fed mice, acetate slightly decreased in FR-fed mice only.

In a previous study, *A. muciniphila* was reported to be decreased in the faecal microbiota of severe asthmatic patients, and oral supplementation of *A. muciniphila* to the native complex gut microbiota was able to modulate the immunophenotype and protect mice from allergic airway inflammation^27^. By contrast, the abundance of *A. muciniphila* or its family Verrucomicrobiaceae has been reported to be transiently increased in the faeces of food allergic infants by the age of 13–18 months^28^. Our results show that *A*. *muciniphila* is lost in sensitized FR-fed mice (Fig. 5c,d); nevertheless, FF-fed mice, which displayed exacerbated food allergy symptoms, did not show a similar complete loss of this bacterium post sensitization (Fig. 5c,d).

### *A. muciniphila* exacerbates food allergic responses under fibre deprivation

**Fig. 6:**
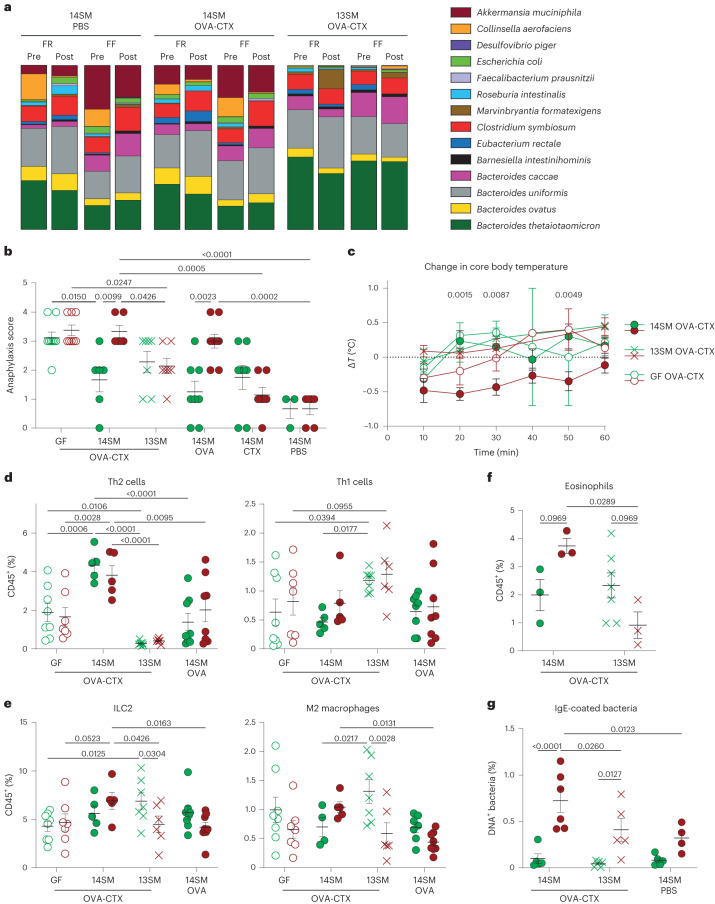
*A. muciniphila* exacerbates type-2 immune responses during food allergy under fibre deprivation in a gnotobiotic mouse model. **a**, Relative abundance of microbial strains assessed from pre- and post-sensitization by phylotype-specific qPCR for both 14SM-colonized (left and middle) and 13SM-colonized (right) mice. **b**,**c**, Blinded symptom scores (**b**) and core body temperature (**c**) acquired after OVA challenge in mice fed an FR (green) or an FF (red) diet (*n* = 3–9, at least two independent experiments, Kruskal–Wallis test with unadjusted *P* values, non-significant after adjustment using the Benjamini–Hochberg method (**b**) or two-way ANOVA (**c**), with *P* values adjusted using the Benjamini–Hochberg method between FR vs FF among OVA-CTX mice). **d**–**f**, Frequencies of immune cell populations identified with mass cytometry and FlowSOM analysis (**d**,**e**, *n* = 5–10, two independent experiments, two-way ANOVA, non-adjusted individual *P* values, bolded P values remain below 0.05 after adjustment using the Benjamini–Hochberg method) and of eosinophils as identified by flow cytometry and FlowJo analysis (**f**, *n* = 3–7, two-way ANOVA, *P* values adjusted using the Benjamini–Hochberg method) in the colonic lamina propria of mice fed an FR or an FF diet. **g**, Frequencies of faecal IgE-coated bacteria at the end of the experiment (*n* = 4–8, two independent experiments, two-way ANOVA, *P* values adjusted using the Benjamini–Hochberg method). All dot plots are represented with mean ± s.e.m.

**Extended Data Fig. 6:**
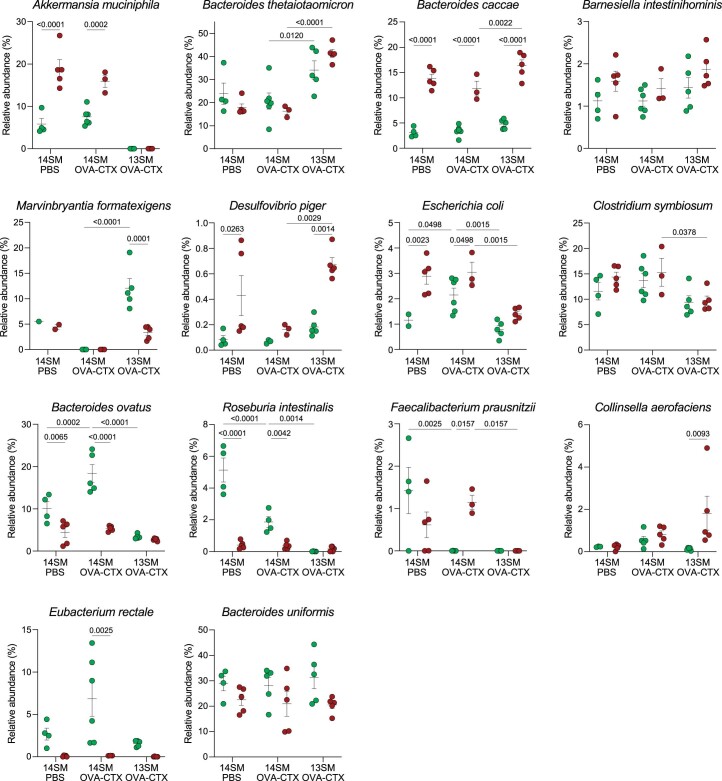
Relative abundances of indicated bacterial strains from feces of FR-fed (green) and FF-fed (red) mice, at the end of the experiment (***n*** = 3–6, two-way ANOVA, ***P*** values adjusted using the Benjamini-Hochberg method). All dot plots are represented with mean +/− SEM.

To investigate the causal role of *A. muciniphila* in modulating food allergy, we used GF mice colonized with a fully characterized, 14-member synthetic human gut microbiota (14SM) in which *A. muciniphila* can be either included (14SM) or excluded (13SM)^14^. In contrast to data in SPF mice (Fig. 5d), *A.* *muciniphila* was still present in FR-fed mice after sensitization, reaching on average 5–11% of the 14SM community (Fig. 6a and Extended Data Fig. 6). Nevertheless, its relative abundance was still increased in FF-fed mice compared with FR-fed mice, reaching on average 13–18% of the community (Extended Data Fig. 6). In the absence of *A. muciniphila*, the relative abundances of the three other mucin-degrading bacteria, *Bacteroides thetaiotaomicron, Bacteroides caccae* and *Barnesiella intestinihominis*, increased in either both dietary groups or in FF-fed mice (Fig. 6a and Extended Data Fig. 6). The low-abundance bacterium *Marvinbryantia formatexigens* and *Desulfovibrio piger* also substantially expanded in the absence of *A. muciniphila*, in FR-fed and FF-fed mice, respectively (Extended Data Fig. 6), whereas the relative abundance of *Escherichia coli*, *Clostridium symbiosum, Bacteroides ovatus, Roseburia intestinalis* and *Faecalibacterium prausnitzii* decreased in either both or one of the 13SM dietary groups (Fig. 6a and Extended Data Fig. 6).

**Extended Data Fig. 7:**
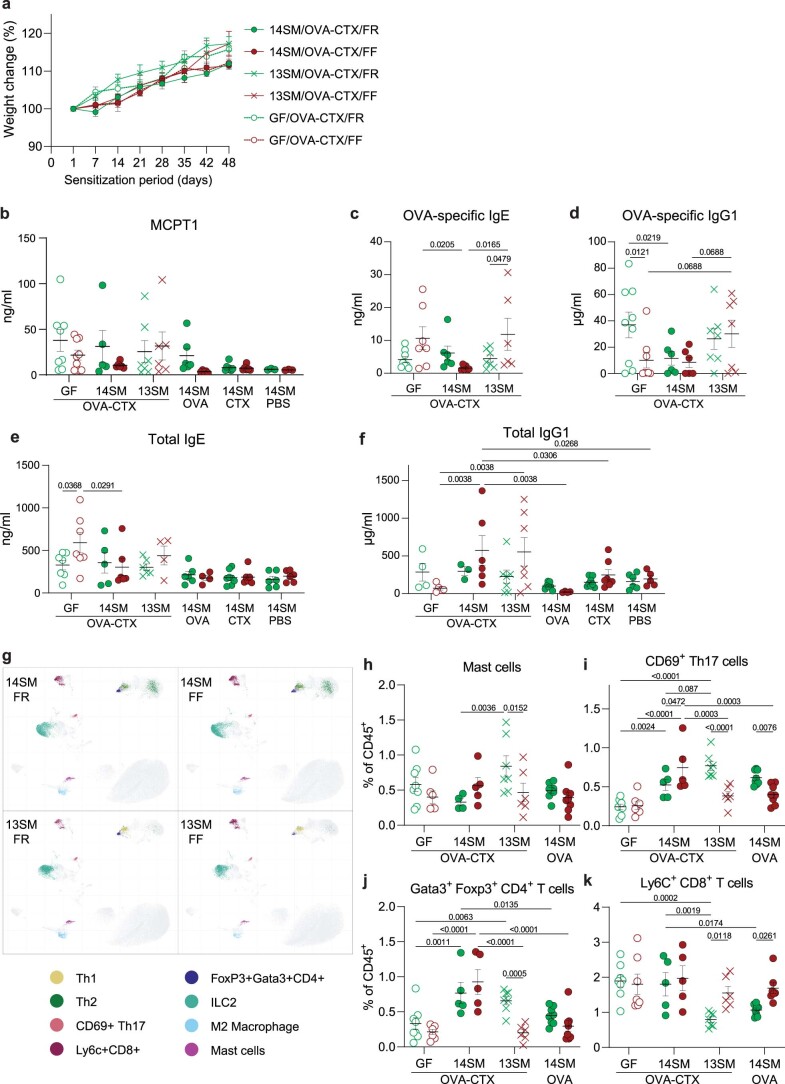
**a**, Weight curves of the mice over the sensitization period to OVA-CTX (n = 3–9, two-way ANOVA with matched values per mice, time effect P < 0.0001). **b–f**, Serum titers of MCPT1 (**b**), OVA-specific IgE (**c**), OVA-specific IgG1 (**d**), total IgE (**e**), and total IgG1 (**f**) (*n* = 4–10 mice per group, two independent experiments, two-way ANOVA, *P* values adjusted using the Benjamini-Hochberg method). Note that the serum titers of OVA-specific IgE and OVA-specific IgG1 were below the limit of detection in other groups. **g**, Uniform manifold approximation and projection (UMAP) of specific populations identified using FlowSOM among CD45^+^ cells from the colonic lamina propria of OVA-CTX sensitized, 14SM- and 13SM-colonized mice 24 h after challenge. Highlighted populations are the ones showing differences between groups. **h–k**, Frequencies of indicated cell populations among cLP CD45^+^ cells (*n* = 5–10, two-way ANOVA, unadjusted *P* values, non-significant after adjustment using the Benjamini-Hochberg method).

As with the SPF mouse data, neither FF feeding nor microbiota composition affected the weight changes in mice during the sensitization period (Extended Data Fig. 7a). In FR diet-fed groups, 14SM-colonized mice treated with OVA-CTX, OVA or CTX did not develop significantly higher symptom scores than PBS control mice, reflective of a moderate model of food allergy symptoms^23^ (Fig. 6b). However, FF-fed OVA-CTX and OVA-treated mice had more severe symptoms than CTX- or PBS-treated mice, in addition to FR-fed OVA-CTX and OVA-treated mice (Fig. 6b), reproducing the same pathological profile as seen with SPF mice (Fig. 3b). These results were corroborated by an increased drop in body temperature in FF OVA-CTX mice as compared with FR OVA-CTX mice (Fig. 6c). As previously reported^7^, GF sensitized FR-fed mice exhibited severe anaphylaxis scores at challenge (Fig. 6b). The GF FF-fed mice showed anaphylaxis scores similar to those of the GF FR-fed mice, suggesting that the FF diet alone, without the microbiota, is incapable of further increasing the allergic severity. When comparing FR-fed OVA-CTX-treated mice, both 13SM- and 14SM-colonized mice had reduced disease scores compared with GF mice, supporting the preventive role of the microbiota in the development of food allergy^7^. By contrast, when the mice were fed an FF diet, only 13SM colonization exhibited decreased symptom scores, while 14SM mice had scores as severe as those of GF mice (Fig. 6b).

High levels of MCPT1 were more frequent in OVA-CTX sensitized mice than in OVA-, CTX- or PBS-treated 14SM-colonized mice (Extended Data Fig. 7b). However and unexpectedly, the titres of OVA-specific Ig showed trends opposite to those of the symptom scores among 14SM- and 13SM-colonized mice (Fig. 6b and Extended Data Fig. 7c,d). While the FF-fed 14SM mice were most affected by the allergen challenge, they had lower titres of OVA-specific IgE and IgG1 than the protected 13SM-colonized mice despite higher titres of total IgE and IgG1 (Fig. 6b and Extended Data Fig. 7c–f), indicating that systemic immunoglobulin titres are not good predictors of the severity of allergic responses.

**Extended Data Fig. 8:**
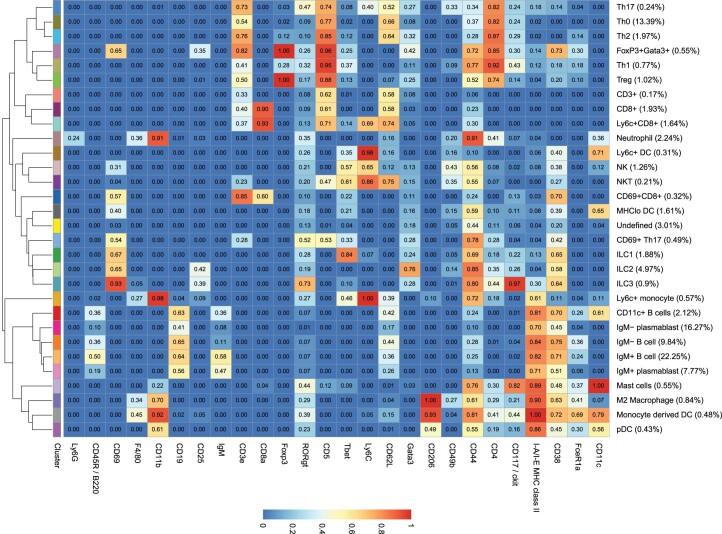
Heatmap of the 30 annotated clusters identified by FlowSOM from CyTOF analysis of colonic lamina propria cells of GF and gnotobiotic OVA-sensitized mice 24 h after challenge. Numbers in the heatmap indicate the relative expression of each marker among cell populations. Percentages shown in parentheses represent frequencies of populations among CD45^+^ cells. All dot plots are represented with mean +/− SEM.

To pinpoint the underlying immune pathways activated by the presence of *A*. *muciniphila* in the microbiota, we performed CyTOF acquisition of the cLP from these mice (Fig. 6 and Extended Data Figs. 7g–k and 8). Interestingly, while sensitized 14SM-colonized mice were characterized by a strong infiltration of colonic Th2 cells, sensitized 13SM-colonized mice had higher frequencies of Th1, suggesting that *A. muciniphila* within the microbiota promotes the recruitment of Th2 cells during sensitization (Fig. 6d). By contrast, innate type-2 cells (ILC2, M2 macrophages and mast cells) and activated CD69^+^ Th17 cells had the tendency to be more abundant in sensitized 13SM-colonized FR-fed mice than in 14SM-colonized FR-fed mice (Fig. 6e and Extended Data Fig. 7h,i). In 13SM-colonized mice, fibre deprivation decreased the abundances of these innate type-2 cells, as well as CD69^+^ Th17 and Gata3^+^Foxp3^+^CD4^+^ T cells, and increased the proportion of Ly6C^+^ CD8^+^ cells (Fig. 6e and Extended Data Fig. 7g–k). In addition, the FF diet increased eosinophil infiltration in 14SM-colonized mice, which was decreased in 13SM-colonized mice (Fig. 6f).

**Extended Data Fig. 9:**
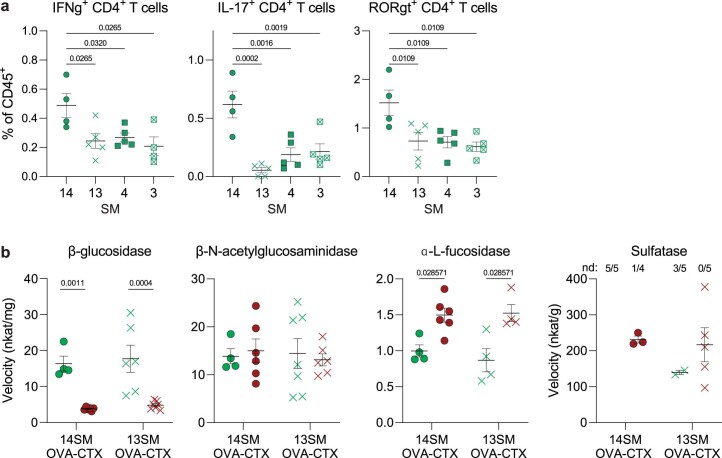
**a**, A figure panel from previously published data by Steimle et al.^29^, showing proportions of indicated cell populations among CD45^+^ cells in the colonic lamina propria of mice colonized with 3SM (*B. caccae*, *B. thetaiotaomicron*, *B. intestinihominis*), 4SM (3SM + *A. muciniphila*), 13SM (3SM + 10 non-mucin-degrading bacteria) or 14SM (13SM + *A. muciniphila*) (*n* = 4–5, one-way ANOVA, *P* values adjusted using the Benjamini-Hochberg method) **b**, Enzymatic activity of select carbohydrate-active enzymes (CAZymes) and sulfatase measured in the feces of OVA-CTX-sensitized, 14SM- and 13SM-colonized mice (*n* = 4–7, multiple Mann-Whitney, *P* values adjusted using the Benjamini-Hochberg method). Fiber-rich (green), fiber-free (red). All dot plots are represented with mean +/− SEM.

These results support a role for *A. muciniphila* in the maintenance of the allergic type-2 response by promoting Th2 cells under fibre-rich conditions and type-2 innate cells under fibre deprivation, while the 13SM community would have a counteracting effect by supporting the Th1 response. Intriguingly, 14SM-colonized mice exposed to OVA only, without adjuvant, had profiles more similar to OVA-CTX sensitized 13SM-colonized mice than to 14SM-colonized ones, with lower levels of innate type-2 cells, CD69^+^ Th17 and Gata3^+^Foxp3^+^CD4^+^ T cells, and more Ly6C^+^ CD8^+^ T cells under fibre deprivation (Fig. 6e and Extended Data Fig. 7g–k). These data suggest that *A. muciniphila* and CTX cooperate to modulate these immune cell populations. In another study using FR-fed C57BL/6 mice^29^, 14SM-colonized mice had more IFNg^+^, IL-17^+^ and RORgt^+^ CD4+ T cells than 13SM-colonized mice, suggesting that the Th1/17 skewing in the absence of *A. muciniphila* might occur following the sensitization (Extended Data Fig. 9a). Furthermore, in FR-fed C57BL/6 mice colonized without the 10 characterized non-mucin-degrading bacteria, the presence (4SM) or absence (3SM) of *A. muciniphila* did not change the proportion of these populations in the colonic lamina propria, suggesting a role for microbial interactions in the regulation of the mucosal immune profile by *A. muciniphila* (Extended Data Fig. 9a). Finally, as shown in SPF mice, FF diet increased the titres of faecal IgE-coated bacteria in OVA-CTX-sensitized mice (Fig. 6g). In the absence of *A. muciniphila* (13SM), this diet effect was reduced (Fig. 6g).

Since *A. muciniphila* is an abundant mucin-degrading bacterium in the 14SM model, we assessed whether its absence in the 13SM community impaired the overall microbial enzymatic activity of carbohydrate-active enzymes (CAZymes) and sulfatases that are known to be involved in mucin foraging (Extended Data Fig. 9b). As expected, the activity of the plant glycan-targeting β-glucosidase was reduced under fibre deprivation in both 13SM and 14SM FF groups as compared with their FR counterparts. In line with the increased relative abundances of other mucin-degrading bacteria in 13SM groups, differences in the mucin-targeting enzyme activities (β-*N*-acetlyglucosaminidase, α-l-fucosidase and sulfatase) between FR and FF groups within 13SM or 14SM communities remained similar.

## Discussion

**Extended Data Fig. 10:**
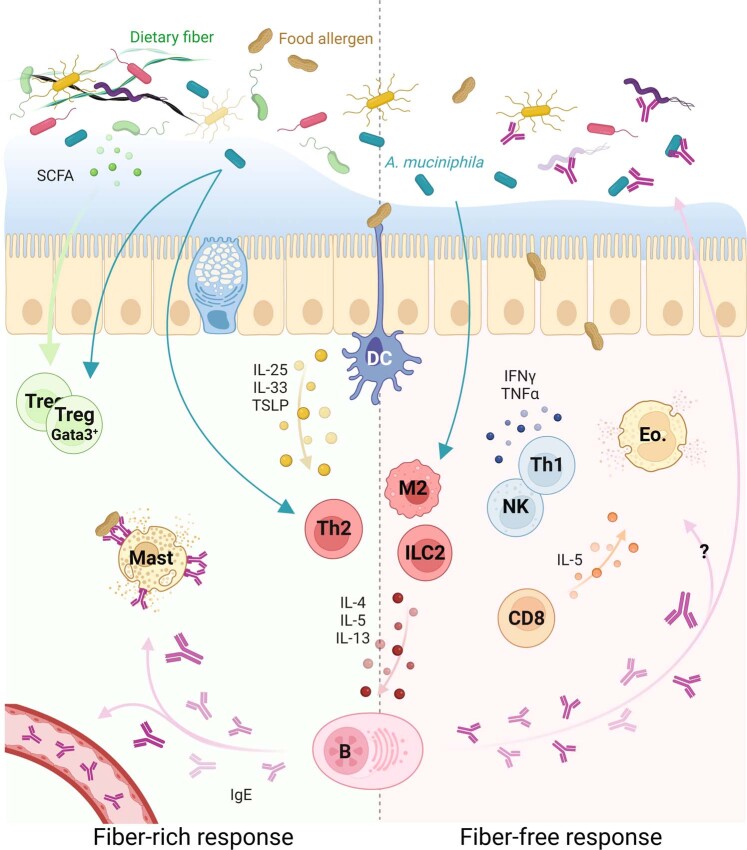
Proposed model of colonic immune pathways during an allergic response under a fiber-rich (left) versus a fiber-free (right) diet. Allergic type-2 responses at the intestinal barrier are initiated by the sensing of the allergen and the release of epithelial-derived cytokines IL-25, IL-33 and TSLP that promote the recruitment and activation of type 2 immune cells Th2, M2 macrophages and ILC2, and lead to the production of IgE. Under a fiber-rich diet, Treg cells are maintained in response to microbiota-derived SCFAs, and *Akkermansia muciniphila* promotes Gata3^+^ Treg cells as well as Th2 cells. In this context, a conventional allergic response is driven by IgE-primed mast cell degranulation. By contrast, fiber deprivation leads to a non-canonical allergy response driven by the altered microbiota composition and the increased intestinal permeability, which altogether predisposes to a mixed inflammatory environment comprising of innate type 2 cells, M2 and ILC2, and type 1 cells, Th1, NK and CD8^+^ T cells. In this context, an allergic response is characterized by the production of IL-5 that is reinforced by CD8^+^ T cells and mediates eosinophilia. This chain of events is paralleled by higher coating of colonic bacteria by IgE, which has an unknown role in the pathology. The fiber deprivation-induced mucus changes and increased intestinal permeability are likely to facilitate the microbiota-driven type 1 responses, but further studies are needed to determine their contribution to the innate type 2 response that is promoted by *A. muciniphila*. Finally, the role of intestinal IgEs and their antigenic specificity in the eosinophilic reaction remain an important question to adress in the future. Created with BioRender.com.

The canonical pathway of food allergy sensitization begins with allergen sensing by dendritic cells and the release of cytokines that promote the recruitment and activation of type-2 immune cells, which leads to the production of IgE (Extended Data Fig. 10). Later, the IgE-primed mast cells degranulate and induce the allergic reaction as soon as they encounter the allergen. Our results suggest that this model is also largely true in the colonic lamina propria, but tunable by dietary fibre. Fibre deprivation leads to a mixed inflammatory environment comprising innate type-2 cells M2 and ILC2, as well as type-1 cells Th1, NK and CD8^+^ T cells. Although present at low frequencies, these are all key effector cells contributing to increase the pool of cytokines such as IL-5 and IFNγ (Fig. 2b,c). The increased secretion of type-2 cytokines, such as IL-5, probably contributes to the eosinophilia and the higher titres of systemic IgE. Interestingly, IFNγ was reported to inhibit mouse mast cell degranulation but potentiate FcƐRI-mediated antigen endocytosis and presentation^30,31^, which may explain the lower titres of MCPT1 despite higher frequencies of mast cells in FF-fed mice before and after sensitization (Fig. 3g and Extended Data Fig. 2c). By contrast, IFNγ can induce piecemeal degranulation of eosinophils^32^. Thus, an immune environment composed of both type-1 and type-2 cells, producing both type-1 and type-2 cytokines, can be seen as an alternative pathway of allergic response in the colon (Extended Data Fig. 10). Intriguingly, allergic gnotobiotic mice recapitulated the symptom profiles of SPF mice, but these were not supported by the systemic titres of OVA-specific IgE. These results beg for an explanation for the role of intestinal IgEs and their antigenic specificity. Although IgE-coated bacteria were recently identified in faecal samples from food allergic children^28^, the mechanism underlying this process is still elusive. Here we report that fibre deprivation can promote the binding of IgE to gut bacteria, even in the absence of experimental allergic sensitization.

*A. muciniphila* has been described as having beneficial health effects, in particular by alleviating mucosal permeability^33^ and the expression of pro-inflammatory cytokines^34,35^. Thus, it is currently proposed as a potential probiotic^36,37^. Our present study shows that the impact of this bacterium is context-dependent and can be detrimental in food allergy when the microbiota is deprived of dietary fibre. In support of our findings, an increase in IL-4 signalling at the transcriptional level in the colon upon monocolonization of GF mice with *A*. *muciniphila* had previously been reported^38^, suggesting a role for type-2 immune skewing in the host. Our results also show that *A. muciniphila* colonization supports non-type-2 immune populations CD69^+^ Th17 and Ly6C^+^ CD8^+^ T cells in the colonic lamina propria of FF-fed and FR-fed sensitized mice, respectively (Extended Data Fig. 7j,k). It is likely that there are other commensal gut microbes that can promote tolerance breakdown following dietary changes, either alone or in combination with other species. Identifying such biomarker species and modulating them with dietary intervention would be important in using gut microbiota as a therapeutic/preventative target in food allergy.

Fibre deprivation increased intestinal permeability in SPF mice, which plays a role in facilitating recognition of food allergens by immune cells. Nevertheless, since the global mucin-degrading capacity of the synthetic community was unchanged in the absence of *A. muciniphila*, these data suggest that increased mucin foraging is not a major driver of the allergic type-2 responses in this model. However, the enhanced mucin-degrading activity of *A*. *muciniphila* in FF mice may be required as an additional microbial trigger to promote the breakdown of oral tolerance, probably through increased innate type-2 responses. Although the Th2 response may be dampened by other unknown changes in the 13SM community, such an effect is nonetheless an indirect consequence of the absence of *A. muciniphila*.

Dietary antigens were long thought to be absorbed in the small intestine, where they promote oral tolerance^39^. In the past decades, however, antigen uptake has been described in the colon through goblet cell-associated antigen passages^40^, suggesting that allergen sensitization may also develop in the colon. Consistently, our data suggest that the colon is a strategic site for diet–microbiota–mucus interactions and food allergic responses. We have previously identified inflammatory effects of the fibre-deprived diet on the colon^22^. Here we present evidence that this inflammatory profile may come as a result of barrier breakdown, allowing greater access of luminal antigens to immune cells. Another study identified a link between acute intestinal infection, which caused a barrier break in the colon, and the subsequent onset of a local dietary antigen (OVA) IgE response^41^. These data suggest that either an infection- or diet-mediated breakdown in the colonic barrier can drive an immunological response in the form of food allergy. This further supports findings among patients suffering from inflammatory bowel diseases and irritable bowel syndrome, who exhibit a high prevalence of food sensitivity^42–44^.

An interesting observation in our broad immunophenotyping data in SPF sensitized mice was the immune skewing for both Th2 and Th1/Th17 that is induced by a fibre-deprived diet. In line with this, in atopic dermatitis, acute inflammation transitioning into a chronic stage is characterized by an overall activation of all Th subsets, rather than Th2 to Th1^45,46^. Allergic inflammation in the fibre-deprived colon can support this phenotype. Overall, our study supports the emerging evidence that food allergy encompasses a vast array of endotypes evolving due to new environmental triggers^3,4,41,47^. Future clinical evaluation and research should focus on immune distinctions and the site of inflammation between food allergic endotypes to design personalized diet- and microbiota-based therapies.

## Methods

### Specific-pathogen-free (SPF) experiments

All SPF animal experiment protocols were approved by the Animal Welfare Service at the Luxembourg Institute of Health and further approved by the Veterinary Services Administration within the Ministry of Agriculture (National Authorization No. LUPA2018/18, LUPA2019/29). Sample sizes were pre-determined with the statistician of the Animal Welfare Service. Six-week-old female BALB/c mice were purchased from Charles River Laboratories. Mice were housed in groups of five and assigned randomly to a diet/treatment group. The FR mice were fed the standard CRM (P) rat and mouse breeder and grower diet (Special Diets Services, 801722). The FF diet was custom-manufactured by SAFE diets according to the TD.140343 diet formulation (Envigo), as previously described^14^. Mice were provided with their respective diets and autoclaved water ad libitum. For readouts before sensitization, mice were fed for 40 d and then euthanized for tissue/sample processing.

### GF and gnotobiotic experiments

All GF and gnotobiotic animal experiment protocols were approved by the Animal Experimentation Ethics Committee at the University of Luxembourg (National Authorization No. LUPA2019/50). The GF status of the mice was verified by culture-based (aerobic and anaerobic) methods. For all gnotobiotic experiments, 6–8-week-old BALB/c mice were housed in isocages in a dedicated axenic room inside a specific opportunistic pathogen-free facility at the University of Luxembourg and were fed the standard diet SAFE R04, irradiated at 40 kGy (SAFE). Mice were colonized with two consecutive daily intragastric gavages of the synthetic microbiota as previously described^48^. One week after the gavage, faecal samples were collected, DNA was extracted and purified, and the colonization of individual strains in the synthetic microbiota was confirmed using phylotype-specifc qPCR primers^48^. Diet was switched for half of the cages to the FF diet (irradiated at 25 kGy, SAFE diets) 10 d after colonization.

### Allergen sensitization and challenge

The ovalbumin sensitization protocol was adapted from ref. ^23^ and was conducted as shown in Fig. 3a. All animals started with a feeding period (40 d for SPF and 7 d for gnotobiotic and GF mice) on either the FR or the FF diet before sensitization. Mice were sensitized by intragastric gavage of 100 μl of the solution related to their treatment group using a reusable 20G feeding needle (FST) once per week for 8 weeks. All solutions were prepared in autoclaved Dulbecco’s phosphate-buffered saline (PBS, LOBE17-515F). Treatment groups were as follows: OVA + CTX (100 µg ovalbumin (A5503, Merck), 10 µg cholera toxin (C8052, Merck)), OVA (100 µg ovalbumin), CTX (10 µg cholera toxin) and PBS (PBS alone). One week after the 8th gavage, the mice were challenged with 5 mg of OVA in 200 μl of PBS. During the 1-h challenge period, two researchers performed blinded core body temperature readouts using a rectal probe (BiosebLab) and recorded the clinical symptom score^23^. Briefly, ‘0’ was assigned if no symptoms were evident; ‘1’ represents mild scratching, rubbing, or both of the nose, head or feet; ‘2’ and ‘3’ represent intermediate symptoms (for example, oedema around the eyes or mouth, piloerection and/or laboured breathing); ‘4’ represents significantly reduced motility, tremors and/or significant respiratory distress; and ‘5’ represents death. Mice were killed 24 h after the allergen challenge, and samples were collected for analysis.

The peanut sensitization protocol was adapted from ref. ^7^ and was conducted as shown in Extended Data Fig. 3a. As with the OVA sensitization protocol, all SPF mice started with a 40-day feeding period on either the FR or the FF diet. All solutions were made up in filter-sterilized Tris (20 mM, pH 7.2). Peanut protein was extracted from defatted peanut flour (Bell Plantation)^7^. Mice were sensitized by intragastric gavage of 400 μl of the solution related to their treatment group: PN + CTX (6 mg PN and 10 µg cholera toxin on days 40 and 42, then 6 mg PN and 15 µg cholera toxin), PN (6 mg PN), CTX (10 µg cholera toxin on days 40 and 42, then 15 µg cholera toxin). On day 75, the mice were challenged twice by gavage with 20 mg of PN, 30 min apart. During the 1-h challenge period, two researchers performed blinded core body temperature readouts using a rectal probe and recorded clinical symptom score^23^. Mice were killed 3 h after challenge and samples were retrieved for analysis.

### Sample processing

All animals were euthanized by cervical dislocation. Blood was extracted immediately by cardiac puncture. Serum was separated by incubating blood samples at 37 °C for 30 min, followed by centrifugation at 845 × *g* for 30 min. Serum was stored at −20 °C for short-term, or −80 °C for long-term storage.

Colons were removed and placed in either methacarn fixative for mucus layer measurements, or collected in HBSS (w/o) (HBSS without Ca^2+^ and Mg^2+^;LOBE10-543F, Westburg) supplemented with 10 mM HEPES (LOBE17-737E, Westburg) for lamina propria cell isolation. Ileal tissues were collected in HBSS (w/o) for lamina propria cell isolation. Both ilea and colons were processed using the lamina propria dissociation kit (130-097-410, Miltenyi Biotec) and gentleMACS dissociator (Miltenyi Biotec) according to the manufacturer instructions.

Caecal, faecal and ileal contents were collected and flash frozen in liquid nitrogen and stored at −80 °C. Caecal, ileal and proximal colonic tissues were cleaned in PBS and stored in 1 ml RNAprotect tissue reagent (76106, QIAGEN) for up to 1 week, followed by long-term storage at −80 °C.

### Mucus layer measurements

After incubation in Methacarnoys fixative^14^, colons were stored in methanol until processing. For processing, samples underwent paraffin embedding and thin longitudinal sections of pellet-containing tissue were set on glass slides. Slides were stained with Alcian blue or anti-MUC2 and quantification was performed in a blinded fashion as previously described^14^.

For MUC2 staining, the slides were deparaffinized in xylene (28973.328, VWR) 3 times for 5 min at room temperature (r.t.). The same 3 consecutive 5 min washes were repeated with pure ethanol (10437341, Fisher Scientific) to dehydrate the sections and then with fresh Milli-Q water to wash the slides. To retrieve the antigens from the tissue sections, Retrievagen A pH 6.0 (550524, BD Biosciences) was heated and immediately poured onto the slides and covered to retain the heat as much as possible. After a 10 min incubation, the slides were left to cool in the solution for 20 min. Then the slides were washed in fresh Milli-Q water 3 times for 5 min, incubated in blocking buffer (Tris-buffered saline (TBS) and 10% goat serum (Gibco, 11540526, Fisher Scientific)) for 1 h at r.t., and gently blotted dry. For staining with the primary antibody, the tissue sections on the slides were covered with the rabbit monoclonal anti-MUC2 antibody (ab272692, Abcam, 1/200) in blocking buffer in the dark for 2 h at r.t. The excess liquid was blotted away and the slides were washed by dipping them into fresh TBS for 5 min for 3 consecutive times. The tissue sections were covered with the secondary antibody Alexa Fluor-488-conjugated goat anti-rabbit IgG (H + L) (10 μg ml^−1^; 10729174, Fisher Scientific) in blocking buffer in the dark for 1 h at r.t. The excess liquid was blotted away and the slides were washed by dipping them into TBS for 3 consecutive times for 5 min. The samples were finally mounted by applying 2–3 drops of ProLong gold antifade mountant with DAPI (Invitrogen, 11549306, Fisher Scientific) and a cover slip to the colon section. After incubating for 24 h at r.t., the slides were sealed using nail varnish and stored in the dark at 4 °C until they were visualized on the Axio Observer Z1 inverted fluorescence microscope (Carl Zeiss). Images were analysed using the MATLAB software BacSpace^15^ to determine mucus layer thickness exclusively in pellet-containing sections. MUC2^+^ goblet cells per crypt were counted by two independent persons using the ImageJ software (https://imagej.nih.gov/).

### Mucus penetrability assay

Penetrability of the colonic mucus was assessed as previously described^20^. Briefly, colons were flushed with ice-cold oxygenated KREBS buffer ‘Transport’ (116 mM NaCl, 1.3 mM CaCl_2_, 3.6 mM KCl, 1.4 mM KH_2_PO_4_, 23 mM NaHCO_3_ and 1.2 mM MgSO_4_; Carl Roth) and opened along the mesenteric axis. The longitudinal muscle layer was removed by blunt dissection and the distal mucosa was inserted in a perfusion chamber. The basolateral chamber was filled with 0.6 µg ml^−1^ SYTO9 (Fisher Scientific, 10237582) in oxygenated KREBS ‘Base’ glucose buffer (KREBS ‘Base’ buffer: 136 mM NaCl, 1.5 mM CaCl_2_, 4.3 mM KCl, 1.6 mM KH_2_PO_4_, 27.1 mM NaHCO_3_ and 1.4 mM MgSO_4_; KREBS glucose buffer: 10 mM glucose, 5.7 mM sodium pyruvate and 5.1 mM sodium-l-glutamate in KREBS ‘Base’ oxygenated buffer; Carl Roth), and the apical chamber was filled with oxygenated KREBS mannitol buffer (containing 10 mM mannitol, 5.7 mM sodium pyruvate and 5.1 mM sodium-l-glutamate in KREBS ‘Base’ oxygenated buffer). After 10 min incubation in the dark at r.t., excess KREBS mannitol buffer was removed and FluoSphere carboxylate beads (1 µm, red 580/605, Invitrogen, F882) were applied on top and allowed to sediment on the tissue for 5 min in the dark at r.t. The apical chamber was then gently washed several times with KREBS mannitol buffer to remove excess beads. The chamber was incubated for 10 min in the dark before being visualized with an Axio Examiner KMAT microscope (Carl Zeiss) using the Zen3.0 (Blue Edition, Carl Zeiss) software. For each tissue, 4–7 confocal images were taken in *XY* stacks from the epithelium at the bottom to the beads on top, with 5-µm intervals between sections. Images were then analysed with Imaris software (Oxford Instruments Imaris). Penetrability was computed as the area under the curve to the peak of bead frequency as a function of the distance from the epithelium (Extended Data Fig. 1c). The same method was used to compute mucus thickness as the distance between the median position of the beads and the median position of the epithelium (Extended Data Fig. 1c,d).

### Intestinal permeability assay

Mice were fasted for 4 h before the assay, followed by oral gavage with FITC-dextran (FD4-250MG, Merck) solution (100 mg ml^−1^ in PBS) so that they received 44 mg FITC-dextran per 100 g of body weight. The mice were anaesthetized 4 h later by intraperitoneal injection of ketamine (12.5 mg per 100 g body weight) and medetomidine (0.025 mg per 100 g body weight), and blood was collected via cardiac puncture in a 1.5 ml tube protected from light. Serum was separated by incubating blood samples at 37 °C for 30 min, followed by centrifugation at 845 × *g* for 30 min. Serum (100 μl) diluted with an equal volume of PBS was added to a 96-well black/clear flat-bottom polystyrene microplate (655906, Greiner). The concentration of FITC in serum was determined by spectrophotofluorometry at an excitation of 485 nm (20 nm band width) and an emission wavelength of 528 nm (20 nm band width), using serially diluted FITC-dextran (0, 125, 250, 500, 1,000, 2,000, 4,000, 6,000, 8,000 ng ml^−1^) as a standard. Serum from mice not administered with FITC-dextran was used to determine the background.

### Bacterial genomic DNA extraction and qPCR analysis

Bacterial DNA was extracted from faecal pellets using phenol-chloroform extraction. Faecal pellets were disrupted in a mix of 500 µl buffer A (200 mM NaCl, 200 mM Tris, 20 mM EDTA), 210 µl 20% SDS, 500 µl equal parts phenol:chloroform and ~250 µl acid-washed glass beads (G1277, Merck) per sample. Mechanical lysis was performed on a Mixer Mill MM 400 (Retsch, Fisher Scientific) for 5 min at 30 Hz, then samples were centrifuged at 18,000 × *g* for 3 min at 4 °C. The aqueous phase was recovered and 500 µl phenol:chloroform was added, mixed by inversion and then centrifuged at 18,000 × *g* for 3 min at 4 °C. The aqueous phase was recovered and 500 µl pure chloroform was added. Samples were mixed by inversion and then centrifuged at 18,000 × *g* for 3 min at 4 °C. The aqueous phase was recovered into a new tube and 1 volume of isopropanol and 1/10 volume 3 M sodium acetate (pH 5.2) were added. Samples were stored at −20 °C for 1 h, followed by centrifugation at maximum speed for 20 min at 4 °C. The supernatants were discarded and the DNA pellets were further cleaned with 1 ml 70% ethanol. Pellets were air-dried for ~1 h and then resuspended in 100 µl of ultrapure water (Invitrogen, 12060346, Fisher Scientific). The DNA was purified using QIAGEN DNeasy blood and tissue kit (69506, QIAGEN) according to manufacturer instructions. Final quantification was performed with a NanoPhotometer N60 (Implen, Fisher Scientific).

DNA (20 ng) was amplified in 12.5 µl of master mix: buffer (10966034, Invitrogen), 1.5 mM MgCl_2_ (10966034, Invitrogen), 200 µM dNTP (10297117, Invitrogen), GelStar nucleic acid gel stain (Lonza, LONZ50535, VWR), 0.5 U Platinum *Taq* DNA polymerase (10966034, Invitrogen) and 0.2 µM each of forward and reverse primers^14^. The qPCR cycle consisted of pre-denaturation at 95 °C for 3 min, followed by 40 cycles of 3 s denaturation at 95 °C, 20 s annealing at 55 °C and 20 s extension at 68 °C. Samples were held at 95 °C for 15 s post-extension and then a melting curve was generated by heating from 60 °C to 95 °C, with 0.3 °C interval increases over 15 s.

### 16S ribosomal RNA gene sequencing and analysis

Bacterial DNA concentrations were measured using a Qubit dsDNA HS assay kit (Q32854, Invitrogen) on a Qubit4 fluorometer (Q33238, Invitrogen). The V4 region of the 16S rRNA gene was amplified using dual-index primers^49^ and sequenced on an Illumina MiSeq system at the Integrated BioBank of Luxembourg (IBBL, Dudelange, Luxembourg). Raw sequences were processed using QIIME 2 (v.2020.6)^50^ with DADA2 for sequence quality control. Sequences were aligned and taxonomic assignment was performed using VSEARCH against the SILVA 138 reference database^51^. A range of 13,769–31,494 reads were obtained per sample (median frequency of 20,029), corresponding to 4,602 features or 345 genera. Data were filtered by removing genera with less than 1 count on average, yielding 214 genera in subsequent analyses. Principal coordinates analysis (PCoA) plots were generated in RStudio v.1.3.1093 (R v.4.0.2) using the package ‘vegan’ (v.2.5-7)^52^, with clustering significance testing using the betadisper() function in vegan and the pairwise.adonis() function in the package ‘pairwiseAdonis’ (v.0.4)^53^ to test for heterogeneity of dispersion and difference in centroids, respectively. Relative abundance plots were generated using the package ‘phyloseq’ (v.1.34.0)^54^ and ‘ggplot2’ (v.3.3.5)^55^. The package ‘DESeq2’ (v.1.30.1)^56^ was used to perform differential abundance analyses.

### RNA extraction

Tissues were thawed on ice in 1 ml of TRIzol reagent (15596026, Life Technologies). A 0.5-mm metal bead (69989, QIAGEN) was added before mechanical lysis on a Mixer Mill MM 400 (Retsch, Fisher Scientific) for 8–10 min at 30 Hz. The extraction was performed according to the TRIzol reagent protocol with a few adaptations. After bead beating, samples were centrifuged at 12,000 × *g* for 5 min at 4 °C. The supernatant was transferred into a fresh tube and 200 µl of pure chloroform was added. Samples were mixed by shaking for 15 s and left to incubate for 3 min at r.t., then centrifuged at 12,000 × *g* for 15 min at 4 °C. The aqueous phase was added to 500 µl isopropanol in a new tube, mixed by shaking for 10 s and left to incubate for 10 min at r.t. The samples were then centrifuged for 10 min at 12,000 × *g* at 4 °C for the precipitation of total RNA. Supernatant was removed and samples were washed with 1 ml cold 75% ethanol. The pellets were left to dry at 37 °C for 5–10 min. Pellets were resuspended in 50 µl nuclease-free water and incubated at 56 °C for 15 min. A DNase treatment was performed following the Thermo Scientific DNase I, RNase-free (EN0521, Thermo Scientific,) protocol, followed by purification with QIAGEN RNeasy mini kit (74106, QIAGEN) according to manufacturer instructions. Final RNA concentrations were quantifed using a NanoPhotometer N60 (Implen). Reverse transcription was performed according to the Invitrogen Superscript IV reverse transcriptase protocol (18090010, Invitrogen) in combination with RNaseOUT (10777019, Invitrogen).

### Cytokine gene expression

qPCR reactions were performed using SYBR Green detection method and Platinum *Taq* DNA polymerase kit (Invitrogen) on a C1000 Touch thermal cycler (Biorad). Before qPCR, complementary DNA samples were diluted 1/5 in 80 µl of nuclease-free water. cDNA (1 µl) was amplified with 11.5 µl of master mix containing buffer (10966034, Invitrogen), 2.5 mM MgCl_2_ (10966034, Invitrogen,), 400 µM dNTP (1029711, Invitrogen), GelStar nucleic acid gel stain (Lonza, LONZ50535, VWR), 0.5 U Platinum *Taq* DNA polymerase (10966034, Invitrogen) and 0.2 µM each of forward and reverse primers. The qPCR cycle consisted of pre-denaturation at 94 °C for 5 min, followed by 40 cycles of 20 s denaturation at 94 °C, 50 s annealing at 60 °C and 45 s extension at 72 °C. Samples were held at 72 °C for 5 min post-extension and then a melting curve was generated by heating from 65 °C to 95 °C with 0.3 °C interval increases over 15 s. mRNA cytokine expression was normalized to the expression of GAPDH. The following primers (Kaneka Eurogentec) were used: *Ifng* forward (5′-ATGAACGCTACACACTGCATC-3′), *Ifng* reverse (5′-CCATCCTTTTGCCAGTTCCTC-3′); *Il22* forward (5′-GCAGCCGTACATCGTCAACC-3′), *Il22* reverse (5′-TCCCCGATGAGCCGGACA-3′); *Il25* forward (5′-ACAGGGACTTGAATCGGGTC-3′), *Il25* reverse (5′-TGGTAAAGTGGGACGGAGTTG-3′); *Il33* forward (5′-AACTCCAAGATTTCCCCGGC-3′), *Il33* reverse (5′-TTATGGTGAGGCCAGAACGG-3′); *Tslp* forward (5′- ACGGATGGGGCTAACTTACAA-3′), *Tslp* reverse (5′- AGTCCTCGATTTGCTCGAACT-3′); *Tnfa* forward (5′-AGCCCACGTCGTAGCAAAC-3′), *Tnfa* reverse (5′-GATAGCAAATCGGCTGACGG-3′); *Il5* forward (5′-AGGCTTCCTGTCCCTACTCAT-3′), *Il5* reverse (5′-TACCCCCACGGACAGTTTGA-3′); *Il17a* forward (5′-TACCTCAACCGTTCCACGTC-3′), *Il17a* reverse (5′-TTCCCTCCGCATTGACACAG-3′); *Il17f* forward (5′-TGAAGTGCACCCGTGAAACA-3′), *Il17f* reverse (5′-GCTACCTCCCTCAGAATGGC-3′); *Muc2* forward (5′-GACGGCGATGTCTACCGATT-3′), *Muc2* reverse (5′-CCAGCTTGTGGGTGAGGTAG-3′); *Gapdh* forward (5′-AATTCAACGGCACAGTCAAGGC-3′), *Gapdh* reverse (5′-GTGGTTCACACCCATCACAAA-3′). Samples showing aberant melting curves were excluded from the analysis.

### CyTOF

To examine the immunological landscape behind the increased allergic phenotype, we performed broad immunophenotyping of the cLP by CyTOF using a 28-marker panel. Single-cell suspension of colonic lamina propria cells were stained with a 28-marker panel as previously described^22^. Briefly, 3 × 10^6^ cells were stained with 5 µM cisplatin solution (201064, Fluidigm) for 5 min. Cells were washed in FACS buffer (PBS, 2% FBS, 2 mM EDTA) and extracellular cell surface staining mix was added for 30 min at r.t. (Supplementary Table 1). Cells were washed twice, fixed using the Foxp3 Transcription Factor Staining buffer set (eBioscience, 00-5523-00, Life Technologies) for 45 min at 4 °C and washed in the permeabilization buffer. The intracellular cell staining mix was added to the cells for 30 min at r.t. (Supplementary Table 1). Samples were washed twice in FACS buffer, resuspended in Cell-ID Intercalator-Ir (201192A, Fluidigm) in MaxPar fixation solution (201192 A, Fluidigm) and refrigerated overnight, or for up to 5 d. Before data acquisition on the Helios mass cytometer (Fluidigm), samples were washed twice in PBS and then twice in deionized water. Samples were resuspended in deionized water at 0.5 × 10^6^ cells per ml, with 10% calibration beads (EQ Four Element Calibration Beads, 201078, Fluidigm). Normalized FCS files were imported in FlowJo v.10.8.1 (BD Life Sciences). The files were cleaned to exclude calibration beads and doublets, and CD45^+^ cells were exported as new files, which were later imported into RStudio (v.1.0.143, R v.3.4.4) using the R package flowcore (v.1.44.2) and FlowSOM (v.2.6.0) for unsupervised analysis, following a previously described pipeline^57,58^. We performed FlowSOM^59^ clustering (Extended Data Fig. 5) and generated a uniform manifold approximation and projection (UMAP)^60^ plot to visualize the cell populations contributing to the immune landscape within the cLP (Fig. 4a).

### Cell stimulation and flow cytometry

Before staining for cytokines, single-cell suspensions from ileal and colonic lamina propria were incubated for 4 h at 37 °C in 5% CO_2_, with lymphocyte activation cocktail (423304, Biolegend) in DMEM (LOBE12-604F, Westburg,) complemented with 10% FBS, 100 U ml^−1^ penicillin/streptomycin (LOBE17-602E, Westburg), glutamine (LOBE17-605E, Westburg), 10 mM HEPES (LOBE17-737E, Westburg) and 0.1% β-mercaptoethanol (M6250, Merck). For eosinophils and cytokine expression analysis, cells were washed with PBS and incubated with Zombie NIR (423105, Biolegend) for 15 min at 4 °C before fixation with Cytofix/Cytoperm solution kit (BD Biosciences). Cells were then washed with FACS buffer, incubated with Fc block (1 μg per 10^6^ cells, 553142, BD Biosciences) for 15 min and stained with the antibody mix (Supplementary Table 1) for 30 min at 4 °C. Samples were finally washed and resuspended in PBS for acquisition on a NovoCyte Quanteon flow cytometer (ACEA Biosciences). FCS files were analysed in FlowJo v.10.8.1 (BD Biosciences). For each reported population, counts were normalized to the LD^−^CD45^+^/Single Cells/Width, SSC-H subset and results are presented as a percentage of CD45^+^ cells.

### Lipocalin-2 ELISA

Faecal samples were resuspended in 500 μl ice-cold PBS with 1% Tween-20, followed by agitation at 2,000 r.p.m. for 20 min at 4 °C on a thermomixer (Eppendorf). Samples were then centrifuged for 10 min at 21,000 × *g* at 4 °C. Supernatants were stored at −20 °C until further analysis. Lipocalin-2 detection was performed using the mouse Lipocalin-2/NGAL DuoSet ELISA (R&D Systems, DY1857, Bio-Techne) according to manufacturer instructions.

### MCPT1 ELISA

Mouse MCPT1 was detected in serum samples using the MCPT1 Mouse Uncoated ELISA kit (88-7503-88, Life Technologies) according to manufacturer instructions, but adapted for 384-well microplates.

### Allergen-specific ELISA

Allergen-specific IgE and IgG1 antibodies were quantified from serum samples by sandwich ELISA. For OVA-specific assays, 20 μl of ovalbumin (A5503, Merck) at 100 ng μl^−1^ in PBS were added to each well of a 384-well microplate (781061, Greiner Bio-One) and incubated overnight at 4 °C. Wells were washed 4 times with 100 μl of wash buffer (1% Tween-20, 154 mM sodium chloride and 10 mM Trisma-base) and blocked with 75 μl of blocking buffer (15 mM Trizma-acetate, 136 mM sodium chloride, 2 mM potassium chloride and 1% (w/v) BSA (bovine serum albumin)) for 2 h at r.t. Undiluted mouse sera were used to determine the OVA-specific IgE antibody concentrations, using the mouse anti-ovalbumin IgE monoclonal antibody (Clone E-C1, 7091, Ams Biotechnology) as serially diluted standard (range 0–1,000 pg μl^−1^) in dilution buffer (DB; 15 mM Trizma-acetate, 136 mM sodium chloride, 2 mM potassium chloride, 0.1% (w/v) Tween-20 and 1% BSA). To determine the OVA-specific IgG1 antibody concentrations, mouse sera were serially diluted from 1/400 to 1/12,800 in DB. The mouse anti-ovalbumin IgG1 monoclonal antibody (Clone L71, 7093, Ams Biotechnology) was used as serially diluted standard (range 0–1,000 pg μl^−1^) in DB. After a washing step performed as described above, 20 μl of diluted standards and samples were added to corresponding wells. The plate was then incubated for 90 min at r.t. The washing step was repeated and 20 μl of detection antibody, either the phosphatase alkaline-conjugated goat anti-mouse IgE (SouthernBiotech, 1110-04, ImTec Diagnostics) or the phosphatase alkaline-conjugated goat anti-mouse IgG1 (SouthernBiotech, 1071-04, ImTec Diagnostics) diluted 1/500 in DB, were added to each corresponding well. The plates were then incubated for 90 min at r.t. Following a last wash, 40 μl of substrate (1x phosphate tablet, S0942, Merck) dissolved in 10 ml of substrate buffer (1 mM 2-amino-2-methyle-1-propanole, 0.1 mM MgCl_2_ × 6H_2_O) were added to each well. After a last incubation step for 60 min at 37 °C, the absorbance was measured at 405 nm using an ELISA plate reader (SpectraMax Plus 384 microplate reader and SoftMax Pro 7 software, Molecular Devices). The OVA-specific IgE or IgG1 antibody concentrations were determined for each sample using the corresponding formulated standard curve.

For PN-specific assays, the same protocol was used with adapted antigen. Peanut protein extracted from defatted peanut flour^10^ (Bell Plantation) and diluted at 12.5 ng μl^−1^ in carbonate-bicarbonate buffer (C3041, Merck) was used for the coating overnight. Mouse sera were diluted 1/10 in DB to determine PN-specific IgE antibody levels and 1/100 to determine PN-specific IgG1 antibody levels. Both detection antibodies (goat anti-mouse IgE-phosphatase alkaline-conjugated and goat anti-mouse IgG1-phosphatase alkaline-conjugated (SouthernBiotech, ImTec Diagnostics)) were diluted 1/1,000 in DB. Absorbance was measured at 405 nm using an ELISA plate reader (SpectraMax Plus 384 microplate reader and SoftMax Pro 7 software, Molecular Devices).

### Total IgE and IgG1 ELISA

Total IgE and IgG1 were quantified from serum samples by sandwich ELISA. The same protocol as for allergen ELISA was used, with adapted reagents. Rat anti-mouse IgE at 60 ng per well or rat anti-mouse IgG1 at 20 ng per well (SouthernBiotech, 1130-01 and 1144-01 respectively, ImTec Diagnostics) was diluted in carbonate buffer and used as capture antibody. Mouse sera were serially diluted from 1/10 to 1/320 to determine the total IgE antibody concentrations, and the mouse IgE isotype control (SouthernBiotech, 0114-01, ImTec Diagnostics) was used as the serially diluted standard in DB (range 0–250 pg μl^−1^). To determine the total IgG1 antibody concentrations, mouse sera were serially diluted from 1/500 to 1/16,000 in DB, and the mouse IgG1 isotype control (SouthernBiotech, 0102-01, ImTec Diagnostics) was used as the serially diluted standard in DB (range 0–2,000 pg μl^−1^). Each detection antibody, either the phosphatase alkaline-conjugated goat anti-mouse IgE (SouthernBiotech, 1110-04, ImTec Diagnostics) or the phosphatase alkaline-conjugated goat anti-mouse IgG1 (SouthernBiotech, 1071-04, ImTec Diagnostics), was diluted 1/500 in DB for the assays. The total IgE or IgG1 concentration was calculated the same way as the OVA-specific IgE/IgG1 concentration.

### SCFA quantification

Metabolomics analysis was performed on flash-frozen caecal content. For each sample, approximately 50 mg of caecal contents was mixed with 500 μl Milli-Q water with 2-ethylbutyric acid. Samples were homogenized using 1.4 mm ceramic beads, centrifuged at 21,000 × *g* for 5 min at 4 °C and run on GC–MS according to ref. ^61^.

### Flow cytometry for Ig-coated bacteria

Quantification of Ig-coated bacteria was performed from frozen faecal samples or tissue contents by flow cytometry. Samples were homogenized in 500 µl ice-cold PBS by mixing at maximum speed on a ThermoMixer for 20 min at 4 °C. Samples were topped up with 500 µl PBS, followed by centrifugation at 100 × *g* for 5 min at 4 °C. The supernatant was passed through a 70-µm strainer (Pluriselect, 43-10070-70, ImTec diagnostics), followed by centrifugation at 10,000 × *g* for 5 min at 4 °C. The pellet was resuspended with 1 ml PBS for measurement of the optical density at 600 nm and quantification of the bacteria. Approximately 10^9^ bacteria were used per staining. Samples were incubated with 500 µl PBS with 5% goat serum (Gibco, 11540526, Fisher Scientific) at 4 °C for 20 min. After centrifugation at 10,000 × *g* for 5 min at 4 °C, pellets were resuspended in PBS with 5% goat serum and the appropriate antibody: FITC-conjugated anti-mouse IgA (Clone mA-6E1, eBioscience, 11-4204-83, Life Technologies), PE-conjugated anti-mouse IgE (Clone RME-1, 406908, Biolegend) or PE-conjugated IgG1 isotype (Clone RTK2071, 400408, Biolegend). After incubating for 30 min at 4 °C, samples were washed in 1 ml PBS. Bacterial pellets were resuspended in 200 µl DNA staining solution (0.9% NaCl in 0.1 M HEPES, pH 7.2) with 1:4,000 SYTO 60 red fluorescent nucleic acid stain (5 mM solution in DMSO, S11342, Invitrogen) and incubated for 20 min at 4 °C. Samples were washed in 1 ml PBS and resuspended in 200 µl PBS for data aquisition on a NovoCyte Quanteon flow cytometer (ACEA Biosciences). FCS files were imported into FlowJo v.10.8.1 (BD Biosciences) for analysis. IgE-coated bacteria were quantified using a negative gate constructed with the corresponding isotype control sample (Extended Data Fig. 2d).

### *p*-nitrophenyl-based enzyme assays

The enzyme assays to quantify the activities of β-glucosidase β-*N*-acetyl-glucosaminidase, α-L-fucosidase and sulfatase in faecal pellets of 14SM and 13SM OVA-CTX mice fed an FR or FF diet were performed following a previously described protocol^62^. Data were excluded in cases where insufficient quantities of protein were extracted from the faecal supernatant (β-glucosidase: *n* = 1 among 13SM OVA-CTX FR-fed mice; α-l-fucosidase: *n* = 3 among 13SM OVA-CTX FF-fed mice; sulfatase: *n* = 2 among 14SM OVA-CTX FF-fed mice, *n* = 2 among 13SM OVA-CTX FR-fed mice and *n* = 2 among 13SM OVA-CTX FF-fed mice). Note that the enzyme activities reported here are not to be considered as absolute enzyme activities but are important for relative comparisons between different groups.

### Statistical analysis

Unless otherwise specifed, all statistical analyses were performed in GraphPad Prism v.8.0 and 9.0. Normality tests were performed to verify the distribution of data. Outliers were removed using the ROUT test (*Q* = 2%). Comparisons were performed using either an unpaired *t-*test, a Mann–Whitney test, a two-way ANOVA or a Kruskal–Wallis test, with *P* values adjusted using the Benjamini–Hochberg method. For each graph, one dot represents one mouse and the number of mice per group (*n*) is indicated in the legend.

### Reporting summary

Further information on research design is available in the Nature Portfolio Reporting Summary linked to this article.

### Supplementary information

Reporting Summary

Supplementary Table 1 List of antibodies used for flow cytometry analysis. Antibodies were used at indicated dilutions for the analysis of cytokine-expressing cells (Panel ‘Cytokines’) or eosinophils (Panel ‘Eosinophils’).

## Data Availability

The raw fastq files from 16S rRNA gene sequencing have been deposited in the European Nucleotide Archive (ENA) at EMBL-EBI under accession numbers PRJEB53451 (https://www.ebi.ac.uk/ena/browser/view/PRJEB53451) and PRJEB51707 (https://www.ebi.ac.uk/ena/browser/view/PRJEB51707). We used the SILVA 138 SSU Ref NR 99 database. The mass cytometry datasets for colonic lamina propria have been uploaded to the FlowRepository database under accession number FR-FCM-Z5G2 (https://flowrepository.org/id/FR-FCM-Z5G2).

## Code availability

The CyTOF analysis pipelines used in this study can be found at https://github.com/DII-LIH-Luxembourg/amuc-allergy.
