# Supplementary material for: Akkermansia muciniphila exacerbates food allergy in fibre-deprived mice
Source: Nat Microbiol. 2023 Sep 11;8(10):1863–79. doi: 10.1038/s41564-023-01464-1 (PMC10522492; doi:10.1038/s41564-023-01464-1)
Supplement: Supplementary file 1 — Reporting Summary [file 41564_2023_1464_MOESM1_ESM.pdf]

nature portfolio

Corresponding author(s): Mahesh S Desai

Last updated by author(s): Jul 26, 2023

Reporting Summary

Nature Portfolio wishes to improve the reproducibility of the work that we publish. This form provides structure for consistency and transparency in reporting. For further information on Nature Portfolio policies, see our Editorial Policies and the Editorial Policy Checklist.

Statistics

For all statistical analyses, confirm that the following items are present in the figure legend, table legend, main text, or Methods section.

n/a	Confirmed	
☐	☒	The exact sample size (n) for each experimental group/condition, given as a discrete number and unit of measurement
☐	☒	A statement on whether measurements were taken from distinct samples or whether the same sample was measured repeatedly
☐	☒	The statistical test(s) used AND whether they are one- or two-sided<br>*Only common tests should be described solely by name; describe more complex techniques in the Methods section.*
☐	☒	A description of all covariates tested
☐	☒	A description of any assumptions or corrections, such as tests of normality and adjustment for multiple comparisons
☐	☒	A full description of the statistical parameters including central tendency (e.g. means) or other basic estimates (e.g. regression coefficient) AND variation (e.g. standard deviation) or associated estimates of uncertainty (e.g. confidence intervals)
☐	☒	For null hypothesis testing, the test statistic (e.g. F, t, r) with confidence intervals, effect sizes, degrees of freedom and P value noted<br>*Give P values as exact values whenever suitable.*
☒	☐	For Bayesian analysis, information on the choice of priors and Markov chain Monte Carlo settings
☒	☐	For hierarchical and complex designs, identification of the appropriate level for tests and full reporting of outcomes
☒	☐	Estimates of effect sizes (e.g. Cohen's d, Pearson's r), indicating how they were calculated

Our web collection on statistics for biologists contains articles on many of the points above.

Software and code

Policy information about availability of computer code

Data collection	No custom software was used for data collection.

Data analysis	No custom algorithms or software were used for data analysis. The analysis pipelines in this study employ tools that have been previously described in published literature and are described below. The code used to analyze these data can be found at https://github.com/DII-LIH-Luxembourg.<br><br>Mass cytometry data analysis: Each organ was analyzed independently following the same analysis pipeline, as previously described by Leonard et al (doi:10.1111/all.14716.). FCS files were normalized with the normalization passport EQ-P13H2303_ver2. FCS files were uploaded into FlowJo™ software version 10.8.1 for cleaning. Unsupervised analysis was carried out in RStudio (version 1.0.143, R version 3.4.4) using the R package 'flowcore' version 1.44.2. Single cells were clustered using FlowSOM. Statistical analysis were performed usingGraphPad Prism version 8.0.0 & 9.0.0 for Windows (GraphPad Software, San Diego, California USA, www.graphpad.com).<br><br>16S rRNA gene sequencing and analysis: Raw sequences were processed using QIIME2 version 2020.6 with DADA2 for sequence quality control and taxonomic assignment was performed using the "classify-consensus-vsearch" method against the SILVA 138 reference database. Further analyses were performed in R version 4.0.2 using the package 'phyloseq' version 1.34.0. PCoA plots were generated using the package 'vegan' version 2.5-7, with clustering significance testing using package 'pairwiseAdonis' version 0.4. Differential enrichment analysis was performed using the package 'DESeq2' version 1.30.1, which implements the Wald test to determine statistical significance (p value adjustment using Benjamini-Hochberg method). Visualizations were generated using 'ggplot2' version 3.3.5 and 'forcats' version 0.5.1.<br><br>Image analysis for mucus penetrability assay: Images were acquired with the software Zen3.0 (Blue Edition, Carl Zeiss Microscopy GmbH) and then analyzed with Imaris software (Oxford Instruments Imaris).<br><br>Goblet cell count: goblets cells per crypts were counted using the ImageJ software (https://imagej.nih.gov/).

For manuscripts utilizing custom algorithms or software that are central to the research but not yet described in published literature, software must be made available to editors and reviewers. We strongly encourage code deposition in a community repository (e.g. GitHub). See the Nature Portfolio guidelines for submitting code & software for further information.

Data

Policy information about availability of data

All manuscripts must include a data availability statement. This statement should provide the following information, where applicable:

- Accession codes, unique identifiers, or web links for publicly available datasets
- A description of any restrictions on data availability
- For clinical datasets or third party data, please ensure that the statement adheres to our policy

The raw fastq files from 16S rRNA gene sequencing have been deposited in the European Nucleotide Archive (ENA) at EMBL-EBI under accession numbers PRJEB53451 (https://www.ebi.ac.uk/ena/browser/view/PRJEB53451) and PRJEB51707 (https://www.ebi.ac.uk/ena/browser/view/PRJEB51707). The mass cytometry datasets for colonic lamina propria have been uploaded to the FlowRepository database under accession number FR-FCM-Z5G2 (https://flowrepository.org/).

Field-specific reporting

Please select the one below that is the best fit for your research. If you are not sure, read the appropriate sections before making your selection.

☒ Life sciences ☐ Behavioural & social sciences ☐ Ecological, evolutionary & environmental sciences

For a reference copy of the document with all sections, see nature.com/documents/nr-reporting-summary-flat.pdf

Life sciences study design

All studies must disclose on these points even when the disclosure is negative.

Sample size	Under the animal protocols, a minimum sample size of 5 mice per group was determined to be sufficient to have at least 90% power to detect statistically significant (p<0.05) changes in mucus layer thickness. As preliminary results were aquired, sample sizes were revised with the readouts of interest, ie. symptom scores at challenge and cytometry results. A sample size of 7 to 10 mice per group was finally approved to be sufficient to have at least 80% power to detect statistically significant (p<0.05) changes in symptom scores.
Data exclusions	In 16S gene sequencing analyses, taxa not observed more than once on average across all samples were removed and the data was rarefied to the minimum library size.
Replication	All experiments were reproduced at least twice in independent batches. We did not detect batch effects.
Randomization	Mice were housed in individual cages with up to 5 mice per cage. Each cage was assigned at random to one of the 2 dietary groups: fiber-rich or fiber-free.
Blinding	Symptom scoring was performed blinded by 2 independent experimenters to avoid subconscious researcher biases.<br>All biological samples were processed and analyzed blindly regarding the experimental groups.

Reporting for specific materials, systems and methods

We require information from authors about some types of materials, experimental systems and methods used in many studies. Here, indicate whether each material, system or method listed is relevant to your study. If you are not sure if a list item applies to your research, read the appropriate section before selecting a response.

Materials & experimental systems

n/a	Involved in the study
☐	☒ Antibodies
☒	☐ Eukaryotic cell lines
☒	☐ Palaeontology and archaeology
☐	☒ Animals and other organisms
☒	☐ Human research participants
☒	☐ Clinical data
☒	☐ Dual use research of concern

Methods

n/a	Involved in the study
☒	☐ ChIP-seq
☐	☒ Flow cytometry
☒	☐ MRI-based neuroimaging

Antibodies

Antibodies used

For CyTOF, all antibodies were ordered from Fluidigm. Batch numbers are listed in order by order date (2018, 2019a, 2019b, 2020):
Rat IgG2b Anti-Mouse CD45 (clone 30-F11) labelled with 89Y; catalog # 3089005B/C; batch # 0581829, 0161909, 0161909, 0622022
Rat IgG2a Anti-Mouse Ly-6G (clone 1A8) labelled with 141Pr; catalog # 3141008B/C; batch # 2751702, 1201823, 1401910, 0132018
Rat IgG2a Anti-MouseCD45R/B220 (clone RA3-6B2) labelled with 144Nd; catalog # 3144011B/C; batch # 2851711, 2851711, 2851711, 2851711
Hamster IgG Anti-Mouse CD69 (clone H1.2F3) labelled with 145Nd; catalog # 3145005B/C; batch # 3511606, 3121831, 2211909, 1322039
Rat IgG2a Anti-Mouse F4/80 (clone BM8) labelled with 146Nd; catalog # 3146008B/C; batch # 3121701, 0211903, 1901904, 2601904
Rat IgG2b Anti-Mouse CD11b (Mac-1) (clone M1/70) labelled with 148Nd; catalog # 3148003B/C; batch # 3171702, 1401907, 1401907, 1401907
Rat IgG2a Anti-Mouse CD19 (clone 6D5) labelled with 149Sm; catalog # 3149002B/C; batch # 0761711, 581828, 1901905, 1901905
Rat IgG2b Anti-Mouse CD25 (IL-2R) (clone 3C7) labelled with 150Nd; catalog # 3150002B/C; batch # 1171712, 081811, 1981902, 3401917
Rat IgG2a Anti-Mouse IgM (clone RMM-1) labelled with 151Eu; catalog # 3151006B/C; batch # 3001411, 1171710, 2681906, 2681906
Hamster IgG Anti-Mouse CD3e (clone 145-2C11) labelled with 152Sm; catalog # 3152004B/C; batch # 3181714, 2971806, 2971806, 0132008
Rat IgG2a Anti-Mouse CD8a (clone 53-6.7) labelled with 153Eu; catalog # 3168003B/C; batch # 1151403, 0101806, 2531901, 2531901
Rat IgG2a Anti-Mouse Foxp3 (clone FJK-16s) labelled with 158Gd; catalog # 3158003A/C; batch # 3461707, 1841813, 2421903, 2421903
IgG1 Anti-Mouse RORgt (clone B2D) labelled with 159Tb; catalog # 3159019B/C; batch # 3181709, 3181709, 3181709, 0912006
Rat IgG2a Anti-Mouse CD5 (clone 53-7.3) labelled with 160Gd; catalog # 3160002B/C; batch # 2791503, 2791503, 2931525, 2791503
IgG1 Anti-Mouse Tbet (clone 4B10) labelled with 161Dy; catalog # 3161014B/C; batch # 3391715, 3391715, 3181901, 3181901
Rat IgG2c Anti-Mouse Ly-6C (clone HK1.4) labelled with 162Dy; catalog # 3162014B/C; batch # 2341706, 1201817, 2461904, 3431915
Rat IgG2a Anti-Mouse CD62L (clone MEL-14) labelled with 164Dy; catalog # 3164003B/C; batch # 2851703, 2851703, 2691906, 2691906
Rat IgG2a Anti-Mouse CD326 (EpCAM) (clone G8.8) labelled with 166Er; catalog # 3166014B/C; batch # 0251601, 0251601, 0251601, 0251601
Rat IgG2b Anti-Mouse Gata3 (clone TWAJ) labelled with 167Er; catalog # 3167007A/C; batch # 3481606, 0391808, 2551902, 0592011
Rat IgG2a Anti-MouseCD206 (MMR) (clone C068C2) labelled with 169Tm; catalog # 3169021B/C; batch # 0711816, 1341903, 2671901, 0282009
Hamster IgG Anti-Mouse CD49b (clone HMa2) labelled with 170Er; catalog # 3170008B/C; batch # 2851710, 3511803, 3511803, 3511803
Rat IgG2b Anti-Mouse CD44 (clone IM7) labelled with 171Yb; catalog # 3171003B/C; batch # 1931725, 1201828, 2461903, 3181915
Rat IgG2a Anti-Mouse CD4 (clone RM4-5) labelled with 172Yb; catalog # 3172003B/C; batch # 1201808, 3391809, 3391809, 3391809
Rat IgG2b Anti-Mouse CD117 (ckit) (clone 2B8) labelled with 173Yb; catalog # 3173004B/C; batch # 0331524, 2631811, 2631811, 0452003
Rat IgG2b Anti-Mouse I-A/I-E (MHC Class II) (clone M5/114.15.2) labelled with 174Yb; catalog # 3174003B/C; batch # 0791514, 2631807, 2631807, 2631807
Rat IgG2a Anti-Mouse CD38 (clone 90) labelled with 175Lu; catalog # 3175014B/C; batch # 0341403, 2041807, 2041807, 2041807
Hamster IgG Anti-Mouse FceR1a (clone 36951) labelled with 176Yb; catalog # 3176006B/C; batch # 2381302, 0381905, 0381905, 0381905
Hamster IgG Anti-Mouse CD11c (clone N418) labelled with 209Bi; catalog # 3209005B/C; batch # 0081812, 1521804, 1521804, 2007297-27

The following antibodies were used for flow cytometry:
Rat IgG2a kappa Anti-Mouse CD4 (clone RM4-5) labelled with BV605, Biolegend (catalog #100548; lot #B279163 – July 2019)
Rat IgG2a kappa Anti-Mouse CD45R/B220 (clone RA3-6B2) labelled with BV650, BD Horizon™/BD Biosciences (catalog #563893; lot #8332815 – May 2019, #0195860 – Sept & Dec 2020)
Rat IgG2b kappa Anti-Mouse CD3 (clone 17A2) labelled with BV711, Biolegend (catalog #100241; lot #B245637 – Nov 2018, #B265870 – Apr 2019, #B330904 – Apr & Jun 2021)
Rat IgG2b kappa Anti-Mouse CD45 (clone 30-F11) labelled with BV786, BD Horizon™/BD Biosciences (catalog #564225; lot #9311117 – Jun 2020, #0177317 – Dec 2020, #1063428 – Jun 2021, # 1175935 – Oct 2021)
Rat IgG2a kappa Anti-Mouse CD8 (clone 53-6.7) labelled with PE-Cy5, Biolegend (catalog #100710, lot #B300603 – Jun 2020)
Rat IgG2a kappa Anti-Mouse IL-17A (clone eBio17B7) labelled with eFluor506, eBioscience™ (catalog #69-7177-82; lot #2029236 – Jul

2019)
Rat IgG1 kappa Anti-Mouse IL-13 (clone eBio13A) labelled with Alexa Fluor 488, eBioscience™ (catalog #53-7133-82; lot #2211046 – Aug 2020)
Rat IgG1 kappa Anti-Mouse TNF alpha (clone MP6-XT22) labelled with PerCP-eFluor710, eBioscience™ (catalog #46-7321-82; lot #1998358 – Jun 2019)
Rat IgG1 kappa Anti-Mouse/Anti-Human IL-5 (clone TRFK5) labelled with PE, BD Pharmingen™/BD Biosciences (catalog #554395; lot #8130811 – Jul 2020)
Rat IgG1 kappa Anti-Mouse IFN gamma (clone XMG1.2) labelled with PE-eFluor610, eBioscience™ (catalog #61-7311-82; lot #2071327 – Jul 2019)
Rat IgG2b kappa Anti-Mouse CD45 (clone30-F11) labelled with PE-Cy7, BD Pharmingen™/BD Biosciences (catalog #552848; lot #8205729 – Apr 2019, #9352096 – Feb 2020)
Rat IgG2a kappa Anti-Mouse Siglec-F (clone E50-2440) labelled with BV786, BD OptiBuild™/BD Biosciences (catalog #740956; lot #226552 – Aug 2020, #1116710 – May 2021)
Rat IgG2b kappa Anti-Mouse CD11b (clone M1/70) labelled with PE-CF594, BD Horizon™/BD Biosciences (catalog #562287; lot #188007 – Oct 2020)

The following antibody was used to stain the mucus:
Rabbit monoclonal anti-MUC2 antibody (clone EPR23479-47, catalog #. ab272692, Abcam, Amsterdam, Netherlands)

The following antibodies were used for ELISA:
Mouse Anti-Ovalbumin IgE (Clone E-C1, catalog #. 7091, Ams Biotechnology, Abingdon, UK)
Mouse anti-ovalbumin IgG1 (Clone L71, catalog # ;. 7093, Ams Biotechnology, Abingdon, UK)
Phosphatase alkaline-conjugated goat anti-mouse IgE (SouthernBiotech, catalog #. 1110-04, ImTec Diagnostics, Antwerp, Belgium)
Phosphatase alkaline-conjugated goat anti-mouse IgG1 (SouthernBiotech, catalog #. 1071-04, ImTec Diagnostics, Antwerp, Belgium)
Rat anti-mouse IgE (SouthernBiotech, catalog #. 1130-01, ImTec Diagnostics, Antwerp, Belgium)
Rat anti-mouse IgG1 (SouthernBiotech, catalog #. 1144-01, ImTec Diagnostics, Antwerp, Belgium)

Validation

Standard, commercially available antibodies and their metal tags were selected in consultation with specialists from Fluidigm and validated on test samples prior to their application in this study. Both fluorescent- and metal-tagged antibodies were titrated according to the manufacturer's recommendation in preliminary experiments in order to determine appropriate concentration.

Animals and other organisms

Policy information about studies involving animals; ARRIVE guidelines recommended for reporting animal research

Laboratory animals

The study involved 6-10 week-old, female Balb/c mice.

Wild animals

The study did not involve wild animals.

Field-collected samples

The study did not involve samples collected from the field.

Ethics oversight

All specific-pathogen-free (SPF) animal experiment protocols were approved by the Animal Welfare Service at the Luxembourg Institute of Health, and further approved by the Veterinary Services Administration within the Ministry of Agriculture (national authorization no. LUPA2018/18, LUPA2019/29). All germ-free (GF) and gnotobiotic animal experiment protocols were approved by the Animal Experimentation Ethics Committee (AEEC) at the University of Luxembourg (national authorization no. LUPA2019/50). Mice were housed in individually ventilated cages (Techniplast Sealsafe Plus GM500 or Allentown Sentry SPP™ Mouse cages in SPF or GF/gnotobiotic facilities, respectively) at 20-24°C with 40-70% humidity, under 12-hour light cycles. Sterile water and diets were provided ad libitum.

Note that full information on the approval of the study protocol must also be provided in the manuscript.

Flow Cytometry

Plots

Confirm that:

☒ The axis labels state the marker and fluorochrome used (e.g. CD4-FITC).

☒ The axis scales are clearly visible. Include numbers along axes only for bottom left plot of group (a 'group' is an analysis of identical markers).

☒ All plots are contour plots with outliers or pseudocolor plots.

☒ A numerical value for number of cells or percentage (with statistics) is provided.

Methodology

Sample preparation

Isolated cells were washed with PBS and incubated with Zombie NIR (Catalogue no. 423105, Biolegend Europe BV, Amsterdam, Netherlands), for 15 min at 4°C, prior to fixation with the Cytofix/Cytoperm solution kit (BD Biosciences, Erembodegem-Aalst, Belgium). Cells were then washed with FACS buffer, incubated with Fc block (1 ug/million cells, catalogue no. 553142, BD Biosciences, Erembodegem-Aalst, Belgium) for 15 min and stained with the antibody mix (Extended data Table 1) for 30 min at 4°C.Samples were finally washed and resuspended in PBS for acquisition.

Instrument

NovoCyte Quanteon Flow Cytometer (ACEA Biosciences).

Software	FCS files were analyzed in FlowJo™ (BD Biosciences)
Cell population abundance	At least 200 000 events were acquired.
Gating strategy	For each reported population, counts were normalized to the LD-CD45+/Single Cells/Width, SSC-H subset and results are presented as a percentage of CD45+ cells.

☒ Tick this box to confirm that a figure exemplifying the gating strategy is provided in the Supplementary Information.
